# Transcriptomic Profiling Identifies Symbiosis-Induced GDSL Lipase Genes Associated with Soybean–Arbuscular Mycorrhizal Symbiosis

**DOI:** 10.3390/ijms27156605

**Published:** 2026-07-24

**Authors:** Shichen Huang, Zhangke Xu, Wuyuan Li, Fuli Xie, Dasong Chen, Youguo Li, Hui Lin

**Affiliations:** State Key Laboratory of Agricultural Microbiology, College of Life Science and Technology, Huazhong Agricultural University, Wuhan 430070, China; 623906504@webmail.hzau.edu.cn (S.H.); m15797637920@163.com (Z.X.); lwy13627058173@163.com (W.L.);

**Keywords:** *Glycine max*, arbuscular mycorrhizal symbiosis, GDSL lipase gene family, lipid metabolism, transcriptome profiling

## Abstract

The symbiotic association between soybean and arbuscular mycorrhizal (AM) fungi enhances phosphorus acquisition, improving crop yield and quality. However, the molecular mechanisms underlying nutrient exchange in this symbiosis remain poorly understood. We performed transcriptomic profiling of *Glycine max* cv. Williams 82 roots across three time points during colonization by *Rhizophagus irregularis* BEG141, revealing distinct transcriptional reprogramming between mycorrhizal and non-mycorrhizal roots. Differentially expressed genes (DEGs) were significantly enriched in pathways related to fatty acid biosynthesis, carbon metabolism, and redox homeostasis. Time-course comparisons further identified DEGs associated with transcriptional regulation, biosynthetic processes, and nitrogen metabolism. Notably, five GDSL lipase genes (*GmGELP6*, *GmGELP29*, *GmGELP140*, *GmGELP141*, and *GmGELP151*) exhibited AM-associated expression patterns and were associated with AM-induced lipid-related transcriptional programs. CRISPR/Cas9-mediated disruption of these genes in soybean transgenic hairy roots altered arbuscule development and was associated with reduced expression of mycorrhiza-inducible fatty acid metabolism genes, suggesting that these genes are associated with normal AM colonization and arbuscule development. This study provides a transcriptomic resource and identifies AM-induced *GmGELP* candidate genes associated with normal soybean–AM symbiosis for future functional and mechanistic studies.

## 1. Introduction

Soybean (*Glycine max*) is an economically important legume crop [1], but its growth and yield are often limited by the presence of organic forms of nitrogen and phosphorus in the soil, which cannot be directly absorbed by plants. Traditional approaches to increasing soybean yield through heavy fertilization are both costly and environmentally detrimental [2,3]. In response, major soybean-exporting countries such as the United States and Brazil have increasingly adopted arbuscular mycorrhizal (AM) and rhizobial inoculants as sustainable alternatives, yielding promising results [4,5]. Therefore, elucidating the mechanisms underlying soybean–AM symbiosis is crucial for promoting agricultural application of AM inoculants and advancing sustainable farming practices [6].

Arbuscular mycorrhizal fungi (AMF) establish symbiotic relationships with approximately 80% of terrestrial plant species. Under phosphorus-deficient conditions, AM fungal spores recognize diffusible signals released by plant roots, triggering hyphal differentiation [7]. Fungal hyphae penetrate the root epidermis and form arbuscules within cortical cells, where nutrient exchange occurs across the periarbuscular membrane. While plants acquire phosphorus from AMF, the host must provide substantial carbon resources to sustain this symbiosis. Phosphate signaling components, such as OsPHR1/2/3, have been implicated in transcriptional regulation during AM symbiosis [8,9], and recent studies have further summarized receptor-mediated signaling and host regulatory networks that coordinate fungal colonization and symbiotic functionality [10]. In addition to sugars, accumulating evidence indicates that lipids, particularly long-chain fatty acids (FAs), serve as a major carbon source supplied by host plants to AMF [11,12]. Recent work has further emphasized that lipids function not only as plant-derived carbon sources but also as components of arbuscule-associated membranes and as important molecules for AM fungal development and reproduction [13]. This discovery has shifted research focus toward understanding lipid biosynthesis and transport pathways in AM symbiosis. In *Medicago truncatula*, key genes involved in fatty acid synthesis, modification, and transport have been systematically characterized, including *KASI/DIS*, *FATM*, *RAM2*, and *STR/STR2*, which encode ketoacyl-acyl carrier protein (ACP) synthase I, acyl-ACP thioesterase M, glycerol-3-phosphate acyltransferase, and periarbuscular membrane-localized ATP-binding cassette (ABC) transporters, respectively, and facilitate lipid transfer to fungal partners [14,15,16,17,18,19].

Transcriptomic approaches have provided important resources for understanding AM symbiosis in several model and crop species. In *Medicago truncatula*, transcriptomic analyses of mycorrhizal roots and arbuscule-containing cortical cells identified AM-induced genes associated with arbuscule development and nutrient exchange [20,21]. In *Lotus japonicus*, transcriptome profiling during AM development revealed broad transcriptional reprogramming involving regulatory networks, transport processes, membrane biogenesis, lipid and amino acid metabolism, and genes shared with root nodule symbiosis [22,23,24]. AM-associated transcriptomic changes have also been reported in rice and tomato, supporting the view that AM colonization induces conserved but host-dependent transcriptional reprogramming across diverse plant lineages [25,26].

Compared with these systems, transcriptomic and functional studies of soybean–AM symbiosis remain relatively limited. Previous soybean studies showed that *GmPT10*, *GmPT11*, and *GmPT7* are induced during mycorrhizal colonization or late symbiotic stages [27], and RNA sequencing (RNA-seq) analysis of soybean roots colonized by different AM fungi identified changes in glucose metabolism and sugar transporter expression, including *GmSWEET6* and *GmSWEET15* [28]. More recently, GmSWEET6 was functionally characterized as an AM-inducible soybean sugar transporter involved in sucrose transport toward AM fungi, further supporting the importance of host carbon allocation during soybean AM symbiosis [29]. In addition, transcriptomic responses have been compared among rhizobial, AM, and dual symbioses in soybean [30], and GmPAP33 was found to participate in arbuscule degeneration through phospholipid hydrolysis [31].

Although these studies have advanced our understanding of phosphate transport, carbohydrate metabolism, and arbuscule turnover during soybean AM symbiosis, the transcriptional dynamics of lipid-related genes during progressive AM fungal colonization remain incompletely understood. Moreover, because soybean can establish both AM symbiosis and nitrogen-fixing root nodule symbiosis, a time-course transcriptome dataset may also provide a basis for comparing conserved and potentially legume-associated transcriptional responses during root symbioses [30,32]. Therefore, building on previous transcriptomic studies of mycorrhizal symbiosis [31,33,34,35], a time-course transcriptome analysis of soybean–AMF symbiosis across representative colonization time points could deepen our understanding of this system and help identify genes associated with AM symbiosis.

GDSL esterases/lipases (GELPs) are a class of hydrolytic enzymes characterized by a unique GDSL motif (GDSxxDxG) and extensively participate in plant growth and development, defense responses, and lipid metabolism [36,37,38,39]. In plant immunity, GELPs regulate lipid metabolism and hormonal signaling in response to pathogenic fungal infections [40]. In lipid metabolism, GELPs participate in cuticle formation and seed oil biosynthesis, and some members have been reported to hydrolyze lipid substrates to produce fatty acids or monoacylglycerols [41,42]. However, despite fatty acids being the primary carbon source supplied by plants to AMF, the specific functional roles of GELPs in plant–AMF symbiosis, especially in soybean AM fungal colonization and arbuscule development, remain poorly understood. Given that soybean is both an important oilseed crop and establishes symbiotic relationships with AMF, investigating AM-induced GELPs in soybean may provide useful insights into lipid-associated processes and arbuscule development during soybean–AMF symbiosis, with potential implications for improving mycorrhizal inoculation efficiency and sustainable soybean production.

In this study, we analyzed transcriptomic profiles of Williams 82 roots under non-mycorrhizal conditions and at representative AM fungal colonization time points. By integrating Gene Ontology (GO) and Kyoto Encyclopedia of Genes and Genomes (KEGG) enrichment analyses, co-expression network analysis, and AM symbiosis-related expression patterns, we identified five AM-induced soybean GDSL lipase genes (*GmGELPs*) as candidate genes associated with soybean–AMF symbiosis. We further validated their expression patterns by quantitative real-time reverse transcription PCR (qRT-PCR) and disrupted these genes in soybean hairy roots using CRISPR/Cas9-mediated gene editing. The edited roots showed impaired AM symbiosis, including reduced mycorrhizal colonization and altered arbuscule development, accompanied by reduced expression of mycorrhiza-inducible fatty acid metabolism-related genes. Thus, this study extends previous transcriptomic observations of AM-responsive lipid-related genes by providing preliminary hairy-root-based evidence that soybean AM-induced GDSL lipase genes are associated with AM colonization, arbuscule development, and lipid-related transcriptional responses. Our findings provide a transcriptomic resource and AM-induced *GmGELP* candidates for future mechanistic studies of soybean–AMF symbiosis.

## 2. Results

### 2.1. Transcriptomic Profiling of Soybean Roots During AM Fungal Colonization

**Figure 1:**
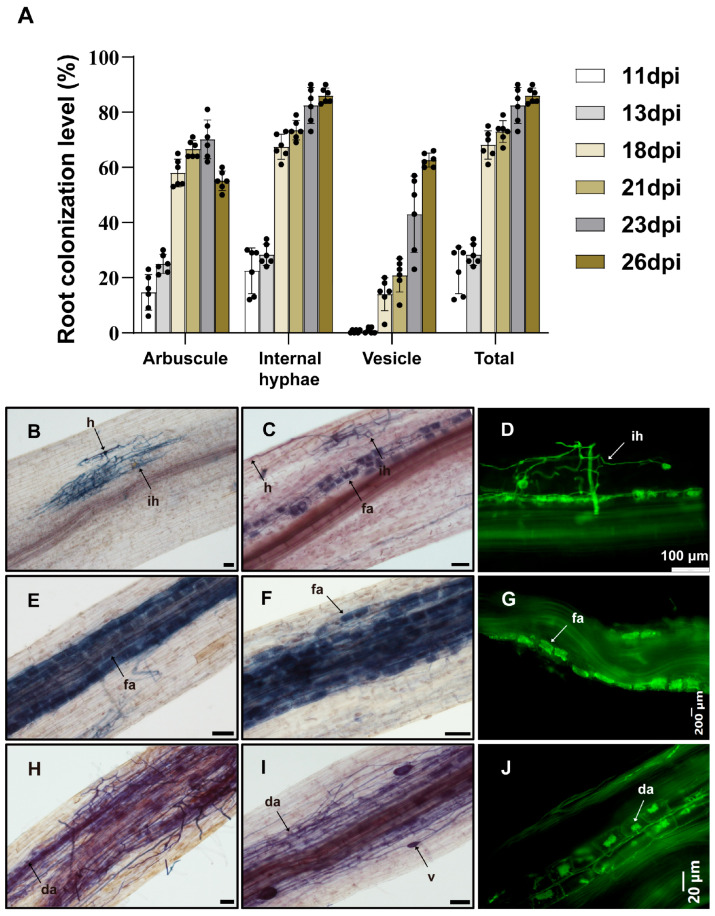
Symbiotic structures in soybean roots at different colonization time points after *R. irregularis* inoculation. (**A**) Fungal colonization levels in soybean roots used for RNA-seq at different time points. Arbuscule, internal hyphae, vesicle, and total colonization levels were quantified from ink-stained roots. Data are presented as mean ± SD. Each dot represents one biological replicate. (**B**–**J**) Representative ink-staining and WGA488 fluorescence images of soybean roots at 13, 21, and 26 dpi after R. irregularis inoculation. Panels (**B**–**D**), (**E**–**G**), and (**H**–**J**) show roots at 13, 21, and 26 dpi, respectively. h, hyphopodium; ih, internal hyphae; fa, fully developed arbuscules; v, vesicle; da, degraded arbuscules; eh, extraradical hyphae. Scale bars are indicated in each panel.

To determine the appropriate sampling time for transcriptomic analysis, we examined soybean roots inoculated with AMF spores across a colonization time course. The mycorrhizal phenotypes were quantified based on total colonization rate, arbuscule abundance, internal hyphal abundance, and vesicle abundance (Figure 1A). At 13 days post-inoculation (dpi), internal hyphae were detected and the first arbuscules had initiated, indicating the establishment of AM fungal colonization (Figure 1B–D). At 21 dpi, abundant mature and highly branched arbuscules were observed (Figure 1E–G). At 26 dpi, AM fungal colonization exceeded 90%, and increased numbers of degraded arbuscules and vesicles were detected (Figure 1H–J). Based on these observations, 13, 21, and 26 dpi were selected as representative time points reflecting increasing AM fungal colonization and changes in arbuscule abundance and morphology. The 21 dpi time point was used for the AM21 vs. NM21 comparison because it showed abundant arbuscule development and was expected to represent active symbiotic nutrient exchange.

Principal component analysis (PCA) was performed based on log_2_(FPKM + 1)-transformed expression values from all RNA-seq samples, where FPKM represents fragments per kilobase of transcript per million mapped reads. The samples were generally grouped according to treatment and sampling time point. NM21 and AM21 biological replicates clustered closely, while AM13 and AM26 showed somewhat greater within-group dispersion. Overall, the four sample groups were distinguishable along the major principal components (Figure S1A).

**Figure 2:**
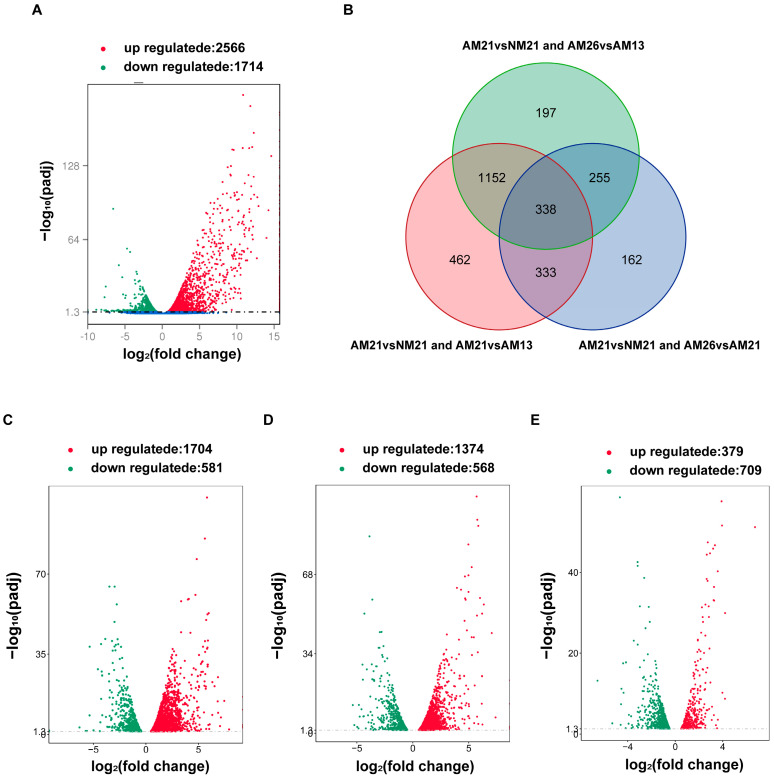
Differentially expressed genes in soybean roots during AM symbiosis. (**A**) Volcano plot showing differentially expressed genes between AM21 and NM21. Red and green dots indicate upregulated and downregulated genes, respectively, with adjusted *p* value (*p*adj) < 0.05 and |log_2_(fold change)| > 0. (**B**) Venn diagram showing the overlap of DEGs among the indicated comparisons. (**C**–**E**) Volcano plots showing DEGs in AM13 vs. AM21, AM13 vs. AM26, and AM21 vs. AM26, respectively. Red and green dots indicate upregulated and downregulated genes, respectively, with *p*adj < 0.05 and |log_2_FC| > 0.

The transcriptomes of soybean mycorrhizal and non-mycorrhizal roots (AM21 vs. NM21) were compared, and differentially expressed genes (DEGs) were analyzed (Supplementary Data Set 3). A total of 4280 DEGs were identified, of which 2566 were significantly upregulated and 1714 were significantly downregulated (Figure 2A).

GO enrichment analysis revealed that upregulated DEGs were significantly enriched in biological processes and molecular functions including fatty acid biosynthesis, transmembrane transport, oxidoreductase activity, iron ion binding, and cellular components such as extracellular region and lysosome (Figure S1B). The downregulated DEGs were predominantly enriched in biological processes such as hydrogen peroxide catabolic process and cell wall organization; molecular functions including peroxidase activity, water channel activity, and inorganic phosphate transmembrane transporter activity; and cellular components such as endoplasmic reticulum lumen and cell wall (Figure S1C).

KEGG enrichment analysis revealed that the upregulated DEGs were predominantly involved in pathways related to fatty acid biosynthesis and metabolism, carbon metabolism (glycolysis/gluconeogenesis and the tricarboxylic acid cycle), nitrogen metabolism (amino acid metabolism), autophagy (lysosome), and terpenoid metabolism (carotenoid biosynthesis) (Figure S1D). The downregulated DEGs were predominantly involved in pathways related to hydrogen peroxide catabolic process, carbon metabolism (starch and sucrose metabolism, amino sugar and nucleotide sugar metabolism), phenylalanine metabolism, and phenylpropanoid biosynthesis (Figure S1E).

### 2.2. Gene Expression Changes Across AM Colonization Time Points

Differential expression analysis using adjusted *p* value (*p*adj) < 0.05 identified 1704 upregulated and 581 downregulated genes in the AM13 vs. AM21 comparison, 1374 upregulated and 568 downregulated genes in the AM13 vs. AM26 comparison, and 379 upregulated and 709 downregulated genes in the AM21 vs. AM26 comparison (Figure 2C–E).

Comparison of DEGs across AM colonization time points revealed substantial overlap between mycorrhizal time-point comparisons and the AM21 vs. NM21 comparison. Venn diagram analysis revealed 2285 shared DEGs between the AM13 vs. AM21 and AM21 vs. NM21 comparisons, 1942 shared DEGs between the AM13 vs. AM26 and AM21 vs. NM21 comparisons, and 1088 shared DEGs between the AM21 vs. AM26 and AM21 vs. NM21 comparisons (Figure 2B).

GO enrichment analysis revealed that genes upregulated in the AM13 vs. AM21 comparison were significantly enriched in processes related to fatty acid biosynthesis, transmembrane transport, and ion transport, whereas downregulated genes were predominantly associated with DNA-templated transcriptional regulation, transcription factor activity, oxidoreductase activity involving nitrogen-containing compounds, and extracellular regions (Figure S2A,B). Genes upregulated in the AM13 vs. AM26 comparison were significantly enriched in fatty acid biosynthesis, transmembrane transport, and fatty acid synthase activity, whereas downregulated genes were predominantly involved in general biosynthetic processes, metabolic regulation, and transcription factor activity (Figure S2C,D). Genes upregulated in the AM21 vs. AM26 comparison were significantly enriched in negative regulation of endopeptidase activity and nitrate transport, whereas downregulated genes were mainly involved in biogenic amine catabolism (Figure S2E,F).

KEGG pathway enrichment analysis showed that genes upregulated in the AM13 vs. AM21 comparison were primarily enriched in fatty acid biosynthesis, fatty acid metabolism, and carbon metabolism, whereas downregulated genes were predominantly associated with phenylalanine metabolism and arachidonic acid metabolism (Figure S3A,B). Genes upregulated in the AM13 vs. AM26 comparison were primarily enriched in fatty acid biosynthesis and other metabolic processes, whereas downregulated genes were predominantly associated with phenylalanine metabolism and glycerophospholipid metabolism (Figure S3C,D). Genes upregulated in the AM21 vs. AM26 comparison were primarily enriched in zeatin biosynthesis, nitrogen metabolism, and galactose metabolism, whereas downregulated genes were predominantly associated with glycerophospholipid metabolism (Figure S3E,F).

### 2.3. Module Gene Analysis in Mycorrhizal Symbiosis

**Figure 3:**
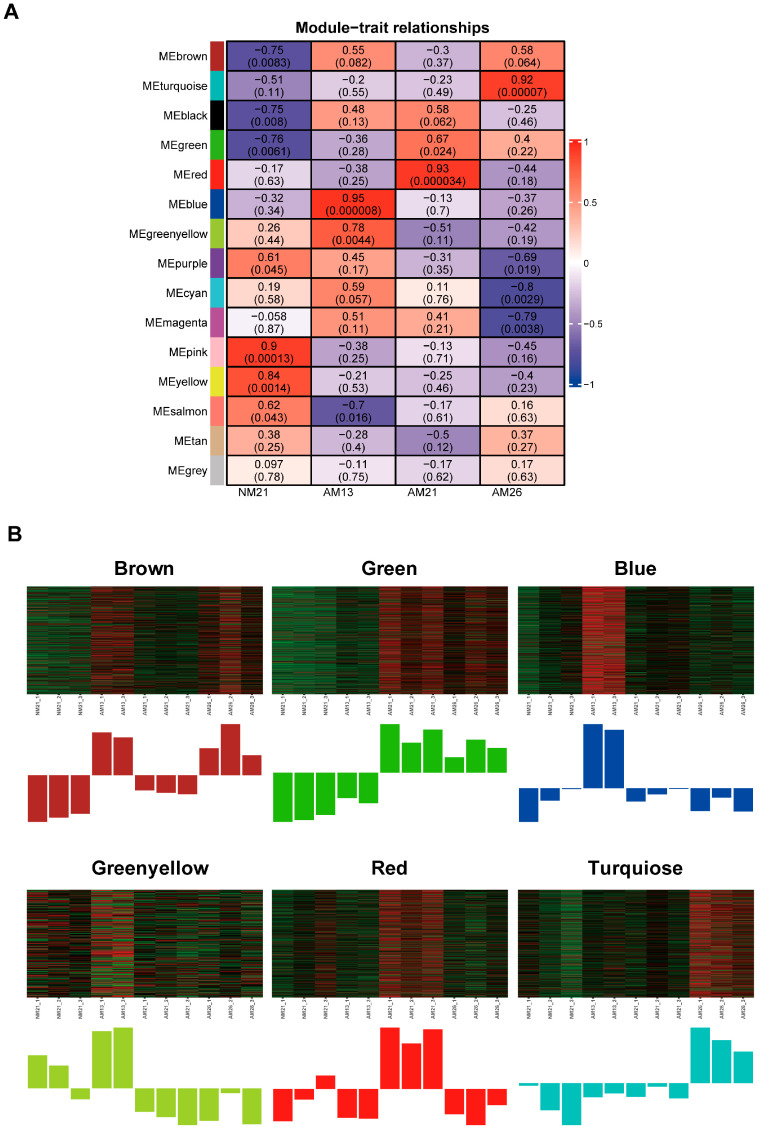
WGCNA module–sample relationships and expression patterns of representative module eigengenes. (**A**) Heatmap showing correlations between WGCNA module eigengenes and sample groups, including NM21, AM13, AM21, and AM26. The color scale represents the correlation coefficient, with red indicating positive correlation and blue indicating negative correlation. Numbers in each cell indicate the correlation coefficient, with the corresponding *p* value shown in parentheses. The grey gene set represents unassigned genes and was not counted as a co-expression module. (**B**) Expression patterns of representative module eigengenes. For each module, the upper heatmap shows the expression patterns of genes assigned to that module across RNA-seq samples, and the lower bar plot shows the corresponding module eigengene values across samples.

To identify gene co-expression patterns associated with AM symbiosis, we performed weighted gene co-expression network analysis (WGCNA). Following the removal of low-expression genes, 16,478 genes were retained, and 15,000 annotated genes were subsequently used for co-expression network construction. A soft-thresholding power of 24 was employed to construct a scale-free network, which yielded 14 co-expression modules (Figure S4A–C). Modules with strong sample associations were defined using the criteria |*r*| > 0.7 and *p* < 0.01, and 11 modules met these criteria (Figure 3A). Here, sample associations refer to correlations between module eigengenes and the four sample groups, NM21, AM13, AM21, and AM26, rather than direct correlations with individual mycorrhizal phenotypic parameters.

To further identify genes associated with AM symbiosis, six representative modules were selected for Gene Ontology (GO) enrichment and expression pattern analysis (Figure 3B). These modules were selected because they showed strong sample associations in the WGCNA, displayed distinct expression patterns across AM colonization time points, and contained GO terms relevant to AM-associated biological processes, including lipid metabolism, autophagy, signal transduction, hormone response, and nitrogen or amino compound metabolism. The brown module genes were predominantly enriched in messenger RNA (mRNA) metabolism and mRNA splicing processes (Figure S5A). Genes in this module were significantly upregulated at AM13 and AM26 and downregulated in the other samples. The green module genes were predominantly enriched in fatty acid metabolism (Figure S5B), with significant upregulation at AM21 and AM26 and downregulation at NM21 and AM13. The blue module genes were predominantly enriched in autophagy and ubiquitin-dependent degradation pathways (Figure S5C), showing significant upregulation specifically at AM13 and downregulation in the other samples. The green-yellow module genes were predominantly enriched in Rab/Ras signal transduction pathways (Figure S5D), with upregulation at AM13 and downregulation at AM21 and AM26. The red module genes were predominantly enriched in endogenous stimulus response and hormone-mediated signaling pathways (Figure S5E), showing significant upregulation at AM21 and downregulation in the other samples. The turquoise module genes were predominantly enriched in organic nitrogen and amino compound anabolism (Figure S5F), showing significant upregulation specifically at AM26 and downregulation in the other samples (Figure 3B).

### 2.4. Co-Expression Network Construction and Identification of Symbiosis-Associated Genes

To identify candidate genes potentially associated with AM symbiosis-related lipid metabolism, a co-expression network was constructed using the six representative modules identified above (Figure S6). Genes located near the center of the network exhibited relatively high connectivity. We then focused on the AM21 vs. NM21 comparison as the primary screening comparison, because AM21 showed abundant arbuscule development and prominent AM-induced transcriptional responses.

A stepwise candidate-selection strategy was used. First, genes significantly induced in AM21 relative to NM21 were considered based on the DEG analysis. Second, genes with relatively high expression abundance and strong AM induction were prioritized, with FPKM values around or above 100 at AM21 and log_2_(fold change) ≥ 6 in AM21 vs. NM21 used as the main prioritization criteria. These thresholds were used only for candidate prioritization, not for DEG identification. Third, previously reported AM markers or functional genes were excluded, because the aim was to identify previously uncharacterized AM-induced candidate genes rather than to re-identify known AM symbiosis markers. Finally, candidate genes annotated as SGNH/GDSL lipase family members were retained for further analysis.

Based on these criteria, four strongly AM-induced GDSL lipase genes showing symbiosis-associated expression patterns were identified: *GmGELP6*, *GmGELP29*, *GmGELP141*, and *GmGELP151* (Figure S7B; Table S2). Previous studies have shown that GDSL lipases constitute a large and functionally diverse gene family in plants [36,37,43]. *GmGELP140* was additionally retained for further analysis despite its lower expression level because it also showed an AM-associated expression pattern (Figure S7B; Table S2). Therefore, these five genes were selected as AM-induced *GmGELP* candidates for expression validation and CRISPR/Cas9-mediated hairy-root functional analysis during soybean AM symbiosis. The public Phytozome gene IDs, coding sequences, and amino acid sequences of these candidate genes are provided in Supplementary Data Set 9.

**Figure 4:**
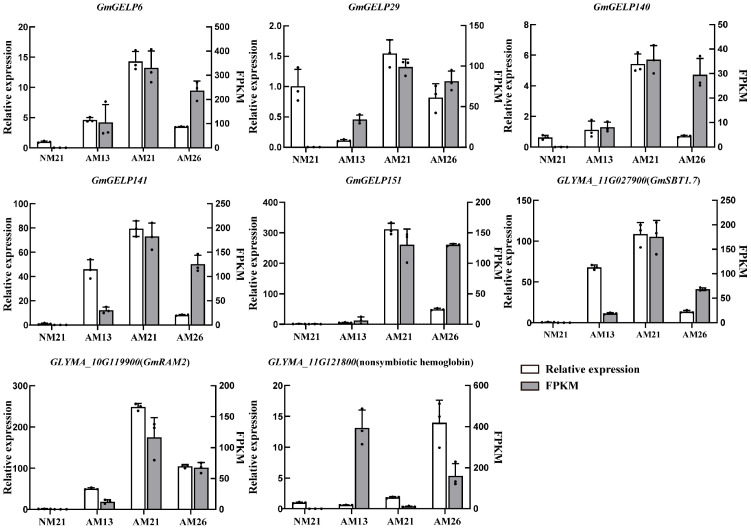
Gene expression levels in soybean roots inoculated with *R. irregularis*. Comparison of qRT-PCR validation and RNA-seq FPKM values for selected AM-associated genes in soybean roots at NM21, AM13, AM21, and AM26. The analyzed genes included *GmGELP6*, *GmGELP29*, *GmGELP140*, *GmGELP141*, *GmGELP151*, Glyma_11G027900 (*GmSBT1.7*), Glyma_10G119900 (*GmRAM2*), and Glyma_11G121800 (nonsymbiotic hemoglobin). *GmSBT1.7*, *GmRAM2*, and Glyma_11G121800 were used as AM-associated validation genes. White bars indicate relative expression levels determined by qRT-PCR, and gray bars indicate FPKM values from RNA-seq. Data are presented as mean ± SD from three biological replicates. The soybean elongation factor gene *GmEF-1α* was used as an internal reference for qRT-PCR.

To further validate the transcriptome results, the expression patterns of the five candidate GDSL lipase genes were examined together with several AM-associated genes. The selected validation genes included GLYMA_11G121800, encoding a nonsymbiotic hemoglobin-like protein; GLYMA_11G027900, encoding a subtilisin-like protein; and GLYMA_10G119900, encoding glycerol-3-phosphate acyltransferase RAM2. The qRT-PCR results were generally consistent with the transcriptome data, supporting the AM-associated expression patterns of these candidate genes (Figure 4).

### 2.5. Structural, Phylogenetic, and Annotation-Based Analysis of GmGELP Proteins

To further elucidate the structural features and chromosomal localization of these genes, conserved-domain analysis of *GmGELP6*, shown as a representative candidate, revealed predicted domains associated with the GDSL/SGNH lipase family (Figure S7A). Amino acid sequence alignment was subsequently performed for the five GmGELP proteins using the amino acid sequences listed in Supplementary Data Set 9. The alignment revealed that their N-termini contain the GDSL-specific motif GDSXXDXG, along with four invariant conserved residues (Ser, Gly, Asn, and His) and the catalytic triad (Ser, Asp, and His) (Figure S8). *GmGELP6*, *GmGELP29*, and *GmGELP151* are located on soybean chromosomes 2, 3, and 16, respectively, while *GmGELP140* and *GmGELP141* are both located on chromosome 15. The latter two have been reported as tandem duplication genes (Figure S9A).

A phylogenetic tree was constructed using soybean GELPs and GELP proteins from other species (Figure S9B). These proteins were classified into 11 subclasses, A–K. This classification is consistent with recent genome-wide phylogenetic analyses showing broad diversification of the GELP family across flowering plants [44]. GmGELP6 and GmGELP151 clustered in subclass D with AtGELP38/51/73/96, which are involved in auxin-mediated lateral root development. GmGELP29, GmGELP140, and GmGELP141 clustered in subclass B near AtGLIP1/2 and TcGLIP, which have been associated with lipid metabolism, pathogen defense, or secondary metabolism. The close genomic relationship between *GmGELP140* and *GmGELP141* as tandem duplicate genes, together with their placement within the same phylogenetic subclass, provided additional support for including *GmGELP140* in the subsequent analyses despite its relatively lower expression level. Together, these structural and phylogenetic features support the annotation of the five GmGELPs as members of the GDSL lipase family and provide clues for their possible functional diversification.

**Figure 5:**
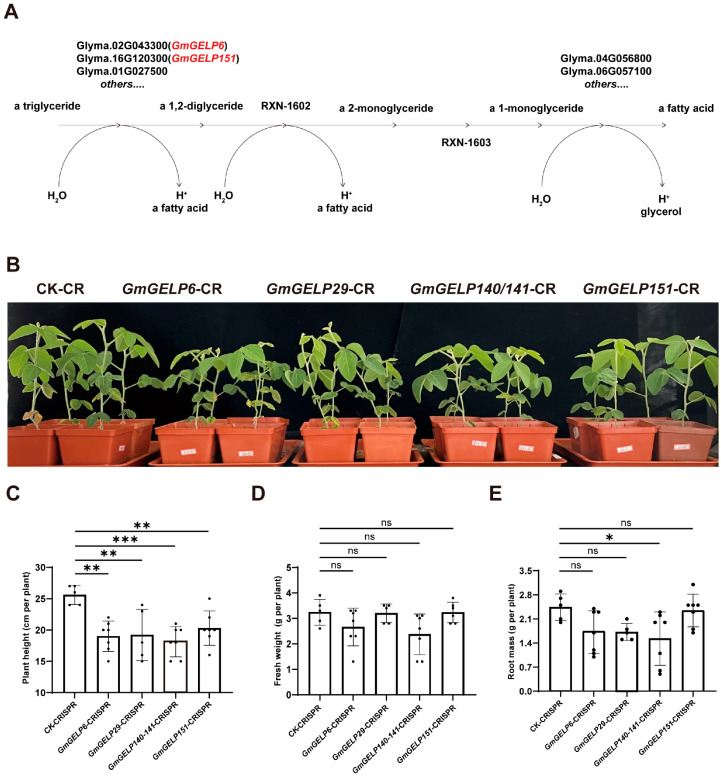
Phenotypes of *GmGELP*-targeting soybean hairy roots. (**A**) Annotation-based prediction of putative lipid hydrolysis-associated processes involving *GmGELP6* and *GmGELP151*. These predictions were based on Phytozome annotation and should be interpreted as functional clues rather than direct evidence of TAG degradation activity. The predicted positions of *GmGELP6* and *GmGELP151* are indicated. (**B**) Growth phenotypes of soybean plants carrying empty-vector control hairy roots or *GmGELP*-edited hairy roots at 4 weeks post-inoculation (wpi). CR, CRISPR/Cas9-edited hairy roots. (**C**–**E**) Plant height, aboveground fresh weight, and root mass, respectively. Data are presented as mean ± SD. Each dot represents one independent soybean plant carrying one GFP-positive transgenic hairy root derived from an independent transformation event. Significance was determined using one-way ANOVA followed by Dunnett’s multiple comparisons test with GraphPad Prism. * *p* ≤ 0.05; ** *p* ≤ 0.01; *** *p* ≤ 0.001; ns, not significant.

Annotation-based predictions from Phytozome suggested that GmGELP6 and GmGELP151 may be related to lipid hydrolysis-associated processes (Figure 5A). Because these predictions are based on sequence annotation, they were used as functional clues for subsequent analysis rather than as direct biochemical evidence.

### 2.6. Effects of GmGELP Disruption on Soybean Mycorrhizal Symbiosis

To examine the effects of *GmGELP* disruption on arbuscular mycorrhizal (AM) symbiosis, CRISPR/Cas9 vectors were assembled (Figure S10A). The single-guide RNA (sgRNA) target sequences, protospacer-adjacent motif (PAM) sequences, on-target scores, target regions, and predicted off-target information are provided in Supplementary Data Set 10. Because *GmGELP140* and *GmGELP141* are tandem duplicated genes, two sgRNAs were designed within a single CRISPR/Cas9 vector to separately target *GmGELP140* and *GmGELP141*. Editing events were verified by polymerase chain reaction (PCR) amplification of target regions from individual green fluorescent protein (GFP)-positive transgenic hairy roots, followed by T-vector cloning and Sanger sequencing of six independent T-clones for each target locus in each root. For each *GmGELP*-targeting construct, three GFP-positive transgenic hairy roots were randomly selected from three independent plants for target-site genotyping. For the *GmGELP140/141* dual-targeting construct, the *GmGELP140* and *GmGELP141* target loci were genotyped separately in each of the same three selected roots, and mutations were detected at both loci in all three genotyped roots. Target-site mutations were detected in all three genotyped *GmGELP*-targeting hairy roots examined, including wild-type (WT)/edited mixed genotypes and edited-only genotypes without detectable WT sequences among the sequenced T-clones (Figure S10B,C). The same genomic DNA samples from the genotyped *GmGELP*-targeting hairy roots were further examined at the predicted off-target sites listed in Supplementary Data Set 10, and no sequence alterations were detected at these tested loci (Figure S10D).

Soybean plants carrying *GmGELP*-targeting hairy roots were shorter than empty-vector control plants (Figure 5B,C), whereas aboveground fresh weight did not differ significantly between genotypes (Figure 5D). Root mass was significantly reduced in *GmGELP140/141*-targeting hairy roots compared to the control, while *GmGELP6*-, *GmGELP29*-, and *GmGELP151*-targeting hairy roots maintained root mass comparable to the control (Figure 5E).

**Figure 6:**
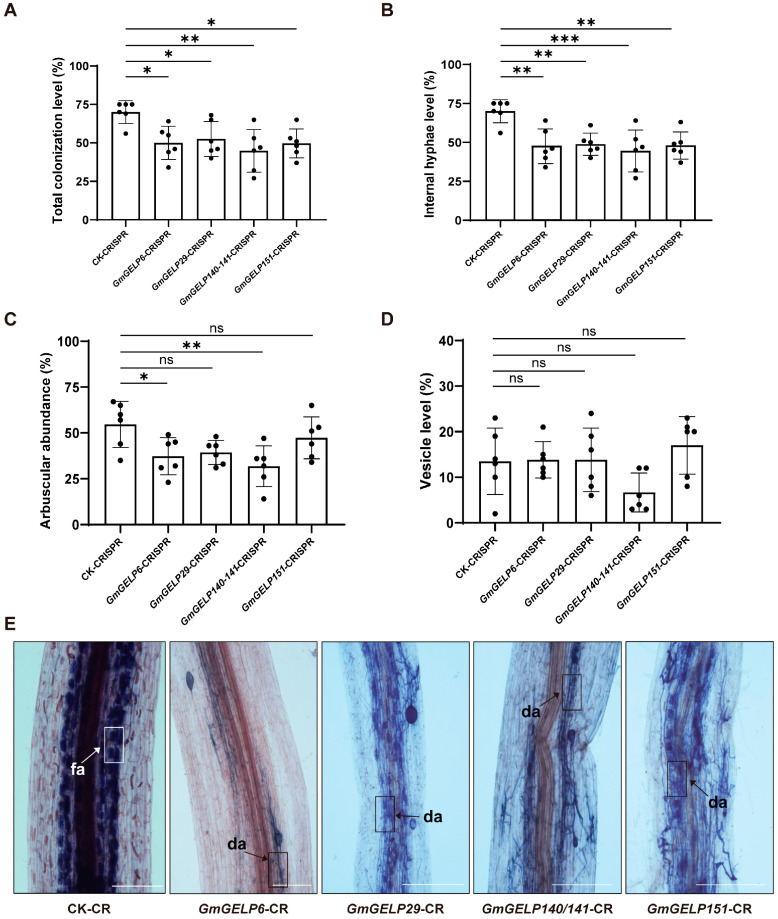
Symbiotic phenotypes of *GmGELP*-edited soybean hairy roots. (**A**–**D**) Comparison of symbiotic phenotypes at 4 weeks post-inoculation (wpi) between empty-vector control hairy roots and *GmGELP*-edited hairy roots. (**A**) Total colonization level. (**B**) Internal hyphal level. (**C**) Arbuscule abundance. (**D**) Vesicle level. (**E**) Representative ink-staining images of soybean hairy roots at 4 wpi, including empty-vector control and *GmGELP*-edited hairy roots. Boxes and arrows indicate representative fully developed arbuscules (fa) in the empty-vector control roots and degraded arbuscules (da) in the *GmGELP*-edited roots. Scale bars = 25 µm. CR, CRISPR/Cas9-edited hairy roots. Data are presented as mean ± SD. Each dot represents one independent GFP-positive transgenic hairy root derived from an independent transformation event. Significance was determined using one-way ANOVA followed by Dunnett’s multiple comparisons test with GraphPad Prism. * *p* ≤ 0.05; ** *p* ≤ 0.01; *** *p* ≤ 0.001; ns, not significant.

The total colonization rate in *GmGELP6*-, *GmGELP29*-, and *GmGELP151*-targeting hairy roots was significantly lower than in controls, decreasing by 20%, 17.5%, and 20.33%, respectively. *GmGELP140/141*-targeting hairy roots showed an even greater decrease of 25.18% (Figure 6A). Hyphal abundance in all *GmGELP*-targeting hairy roots was significantly decreased compared to controls (Figure 6B). Arbuscule abundance in *GmGELP29*- and *GmGELP151*-targeting hairy roots showed a slight decrease. *GmGELP6*-targeting hairy roots showed a significant decrease of 17.37%, while *GmGELP140/141*-targeting hairy roots exhibited a significant decrease of 22.84% compared to controls (Figure 6C). Vesicle abundance did not differ significantly among genotypes (Figure 6D). Moreover, representative images showed degraded arbuscules in *GmGELP*-targeting hairy roots (Figure 6E).

### 2.7. Expression of Fatty Acid Metabolism-Related Genes in GmGELP-Targeting Hairy Roots

**Figure 7:**
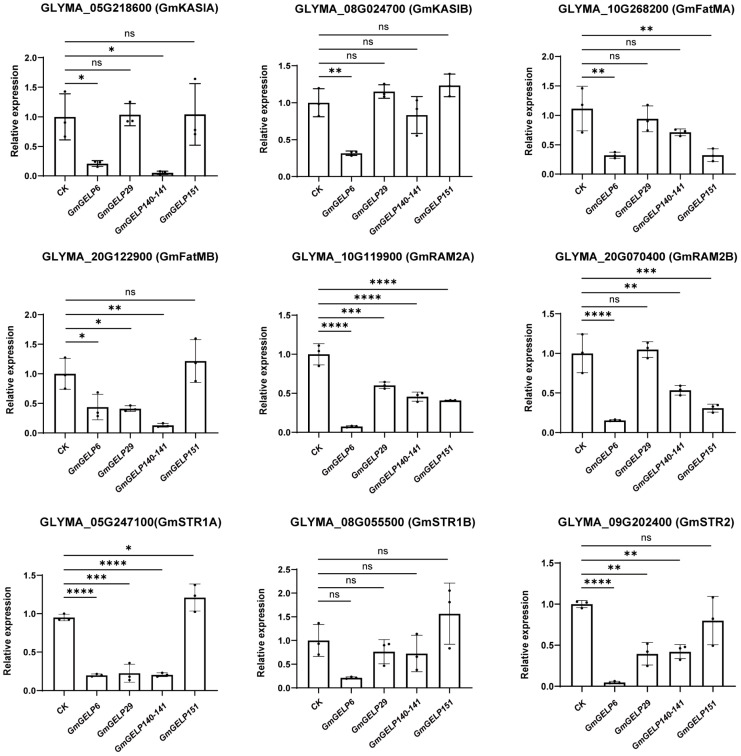
Transcriptional levels of fatty acid metabolism-related genes in GmGELP-edited soybean hairy roots. Expression levels of fatty acid metabolism-related genes in empty-vector control and GmGELP-edited soybean hairy roots were analyzed by qRT-PCR. CK indicates empty-vector CRISPR control; *GmGELP6*, *GmGELP29*, *GmGELP140/141*, and *GmGELP151* indicate the corresponding *GmGELP*-edited hairy roots. The soybean elongation factor gene *GmEF-1α* was used as an internal reference. Data are presented as mean ± SD. Each dot represents one independent GFP-positive transgenic hairy root derived from an independent transformation event. Asterisks indicate significant differences compared with the CK control. Significance was determined using one-way ANOVA followed by Dunnett’s multiple comparisons test with GraphPad Prism. * *p* ≤ 0.05; ** *p* ≤ 0.01; *** *p* ≤ 0.001; **** *p* ≤ 0.0001; ns, not significant.

To investigate whether disruption of these genes was associated with changes in lipid-related transcriptional responses during AM symbiosis, the expression levels of mycorrhiza-inducible fatty acid metabolism-related genes were analyzed in *GmGELP*-targeting hairy roots. Compared to the control, fatty acid metabolism genes were significantly downregulated in *GmGELP6*- and *GmGELP140/141*-targeting hairy roots. In the *GmGELP29*- and *GmGELP151*-targeting hairy roots, some fatty acid metabolism genes exhibited significant downregulation, whereas others showed no consistent trend (Figure 7). These results were consistent with the phenotypic and symbiotic characteristics of the *GmGELP*-targeting hairy roots, indicating that disruption of the five *GmGELPs* is associated with altered expression of AM-inducible fatty acid metabolism-related genes during soybean mycorrhizal symbiosis. However, because reduced colonization and altered arbuscule development may themselves affect the expression of AM-responsive lipid-related genes, the reduced expression of these genes may be a secondary consequence of impaired AM colonization or arbuscule development rather than direct evidence that *GmGELPs* directly regulate fatty acid metabolism-related genes.

## 3. Discussion

The identification of *GmGELPs* (*GmGELP6/29/140/141/151*) as AM-induced genes in soybean provides new candidates for studying the molecular processes associated with soybean–AM symbiosis. These genes belong to the GDSL lipase family and were induced during AM symbiosis. Because host-derived lipid biosynthesis and transfer are central to AM symbiosis [11,12], the AM-associated expression of these *GmGELPs* suggests a possible connection with lipid-related processes during arbuscule development. However, their precise biochemical functions, substrates, and cellular sites of action remain to be determined.

GDSL lipase genes (GELPs) have been implicated in plant cuticle formation, lipid-related metabolism, defense responses, and reproductive development [36,37,40,45,46,47,48,49]. Representative members, including Arabidopsis AtCDEF1 and CUS1, rice OsGLIP1/2, and maize ZmMS30, illustrate the functional diversity of this family. However, the enzymatic and biological functions of most GELPs remain incompletely characterized, particularly in arbuscular mycorrhizal symbiosis.

Although GDSL-like lipases have been reported as AM-responsive genes in some transcriptomic datasets [50,51], functional evidence linking soybean GELPs to AM symbiotic development remains limited. The present study therefore differs from previous descriptive transcriptomic studies by combining time-course soybean–AMF transcriptome profiling with co-expression-based candidate gene identification, qRT-PCR validation, and CRISPR/Cas9-mediated hairy-root editing. This integrated approach revealed associations between AM-induced *GmGELPs*, mycorrhizal colonization, arbuscule development, and lipid-related transcriptional responses in soybean.

Enrichment analysis of AM21 versus NM21 showed that upregulated genes were mainly related to fatty acid and carbon metabolism and redox reactions, whereas downregulated genes were associated with water and phosphorus transport, hydrogen peroxide decomposition, and peroxidase activity. These patterns are broadly consistent with AM transcriptomic studies in other hosts and with the established importance of host-derived lipid biosynthesis and transport for arbuscule development and AM fungal colonization [11,12,14,20,21,22,23,24,25]. Recent evidence from apple roots further supports the importance of lipid-related programs during AM development [52].

Across AM colonization time points, DEGs were enriched in transcriptional regulation, biosynthesis, transport, nitrate transport, and nitrogen metabolism, indicating broad transcriptional reprogramming during colonization. Because soybean forms both AM symbiosis and nitrogen-fixing root nodule symbiosis, the nitrogen-related terms may reflect legume-associated root responses. However, these transcriptomic data do not demonstrate direct regulatory crosstalk between AM symbiosis and nodulation [30,32].

The enrichment of downregulated DEGs in cell wall organization-related terms may reflect the dynamic and cell type-dependent nature of cell wall remodeling during AM symbiosis. Fungal entry and arbuscule formation require local cell wall modification, whereas excessive defense-associated wall reinforcement may restrict symbiotic development. Therefore, the downregulation of some cell wall-related genes in AM21 compared with NM21 may indicate suppression of broad defense- or reinforcement-associated wall responses to maintain a compatible intracellular symbiotic interface. Because our RNA-seq data were generated from bulk roots, these patterns may also reflect the mixed transcriptional signals of colonized and non-colonized cells in roots containing abundant mature arbuscules. This interpretation is consistent with recent spatial co-transcriptomic and cellular-resolution studies showing that AM symbiosis involves discrete spatial and developmental transcriptional states rather than uniform transcriptional responses across all root cells [53,54].

Within the composite-plant hairy-root system used in this study, phenotypic analysis of *GmGELP*-targeting hairy roots provides preliminary evidence that these genes are associated with normal soybean–AM symbiotic development. Compared with the empty-vector control, *GmGELP*-targeting hairy roots exhibited reduced AMF colonization and altered arbuscule morphology. These observations support an association between *GmGELP* targeting and impaired mycorrhizal phenotypes but do not establish that these genes directly determine arbuscule development. Although aboveground fresh weight did not differ significantly among the groups, soybean plants carrying *GmGELP*-targeting hairy roots showed reduced plant height, and root mass was significantly reduced in the *GmGELP140/141*-targeting group. These changes suggest that altered plant growth or root physiology may partly contribute to the observed mycorrhizal phenotypes.

Changes in the expression of fatty acid metabolism-related genes were observed together with impaired mycorrhizal phenotypes in *GmGELP*-targeting hairy roots. Because reduced colonization and altered arbuscule development may themselves affect the expression of AM-inducible lipid metabolism-related genes, these transcriptional changes may represent secondary consequences of impaired symbiosis and should not be interpreted as evidence that *GmGELPs* directly regulate lipid metabolism. Although annotation-based predictions suggest that some *GmGELPs* may be related to lipid hydrolysis-associated processes, these predictions should be interpreted as functional clues rather than direct evidence of enzyme activity, substrate specificity, or cellular localization.

Given the functional diversity of the GELP family, the biological roles of *GmGELPs* cannot be inferred solely from sequence similarity or transcriptional induction. Therefore, although five GmGELPs showed AM-associated expression patterns and *GmGELP*-targeting hairy roots displayed altered mycorrhizal colonization and arbuscule development, we interpret them as AM-induced *GmGELP* candidates associated with normal mycorrhizal development rather than as direct determinants of arbuscule development or biochemically defined lipid-metabolic enzymes. Establishing a definitive causal role will require complementation or re-expression experiments and stable whole-plant validation. Whether homologous *GELP* genes are also induced during AM symbiosis in other host species, such as *Medicago truncatula*, *Lotus japonicus*, and rice, remains to be determined.

Several limitations of this study should be noted. First, this study did not directly measure lipid content, fatty acid transport, lipid composition, or GmGELP enzyme activity; therefore, the direct substrates, biochemical activities, and precise roles of *GmGELPs* in lipid metabolism remain unknown. Second, potential functional redundancy among soybean GELP family members was not systematically evaluated. Although *GmGELP140* and *GmGELP141* were targeted together because they are tandem duplicate genes, other closely related or uncharacterized *GmGELPs* may partially compensate for the loss or reduction in function of the targeted genes. Such compensation could attenuate the observed phenotypes and limit our ability to assign nonredundant, gene-specific roles to individual *GmGELPs*. Future studies using higher-order mutants, complementation or re-expression experiments, and stable whole-plant materials will be required to evaluate the extent of functional redundancy within this gene family.

Third, the CRISPR/Cas9 functional analyses were conducted using a soybean composite-plant hairy-root system, in which a transgenic hairy root develops on a plant with a non-transgenic shoot. Although this system enables rapid root-specific functional analysis, it does not fully reproduce the systemic coordination between roots and shoots or the developmental and physiological context of a stably transformed whole plant. Therefore, the observed changes in AM colonization, arbuscule morphology, root growth, and shoot growth should not be directly extrapolated to stable whole-plant mutants. Moreover, the experiments were conducted under controlled growth conditions, which may not fully represent soybean–AMF interactions in natural soils or field environments. Validation using stable whole-plant materials and experiments under more natural symbiotic conditions will be necessary to determine the generality of these findings.

Fourth, complementation or re-expression experiments were not performed. Although the predicted off-target site listed in Supplementary Data Set 10 for each sgRNA was examined by Sanger sequencing and no mutations were detected at the tested loci, genome-wide or deep amplicon sequencing-based off-target assessment was not performed. Therefore, potential off-target effects cannot be completely excluded.

Fifth, the spatial expression patterns of *GmGELPs* were not determined by promoter–reporter assays or in situ hybridization; therefore, whether these genes are specifically expressed in arbuscule-containing cortical cells remains to be determined. Finally, the observed lipid-related transcriptional changes may be secondary to altered root growth, reduced AM colonization, or abnormal arbuscule development and therefore do not demonstrate direct regulation of lipid metabolism by *GmGELPs*. Future studies combining complementation or re-expression, whole-plant validation, off-target assessment, cell-type-specific expression analysis, lipidomic profiling, and enzyme activity assays will be required to define the direct molecular roles of *GmGELPs* during soybean–AMF symbiosis.

**Figure 8:**
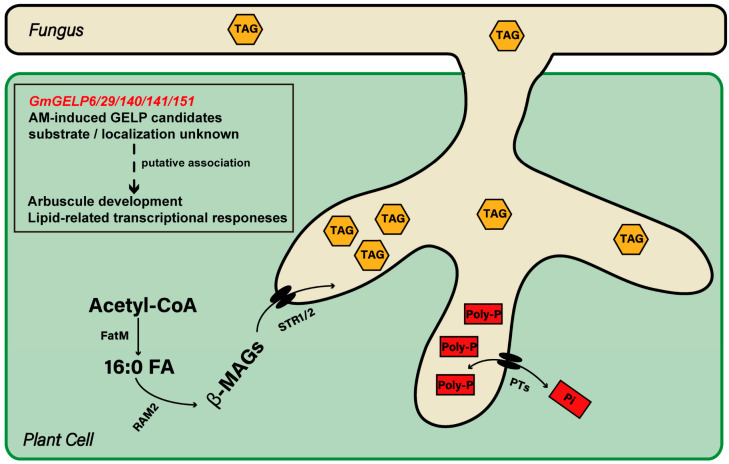
Proposed model summarizing the association between GmGELPs and soybean mycorrhizal symbiosis. The model summarizes the association between *GmGELPs*, arbuscule development, and lipid-related transcriptional responses during soybean arbuscular mycorrhizal (AM) symbiosis. *GmGELPs* indicate soybean GDSL esterase/lipase genes. Solid arrows indicate previously established AM lipid biosynthesis and transfer pathways, including FatM-, RAM2-, and STR1/2-associated steps. FatM indicates acyl-acyl carrier protein thioesterase M; RAM2 indicates REQUIRED FOR ARBUSCULAR MYCORRHIZATION 2; STR1/2 indicates STUNTED ARBUSCULE 1/2. TAG indicates triacylglycerol; FA indicates fatty acid; 16:0 FA indicates a 16-carbon saturated fatty acid; β-MAGs indicate β-monoacylglycerols. PTs indicate phosphate transporters; Pi indicates inorganic phosphate; Poly-P indicates polyphosphate. Dashed arrows indicate putative or indirect associations inferred from *GmGELP* expression patterns, edited-root phenotypes, and lipid-related gene expression changes. The biochemical substrates and subcellular localization of *GmGELPs* remain to be determined.

In conclusion, this study describes differential gene expression profiles of soybean roots across three AM fungal colonization time points and identifies five AM-induced GmGELP candidate genes associated with normal soybean–AM symbiosis. The hairy-root results support associations between *GmGELP* targeting, altered arbuscule development, and lipid-related transcriptional responses, but do not establish that these genes directly regulate arbuscule development or lipid metabolism. Accordingly, these *GmGELPs* should be regarded as promising candidates for further functional study rather than as confirmed regulators of AM symbiosis. Based on these findings, we propose a working model summarizing the observed associations during soybean AM symbiosis (Figure 8). Definitive functional characterization will require complementation or re-expression analyses, validation in stable whole-plant materials, evaluation under natural or field-relevant symbiotic conditions, and further biochemical and molecular investigation of their substrates, activities, and cellular functions.

## 4. Materials and Methods

### 4.1. Plant Materials, AM Fungal Inoculation, and RNA-Seq Sampling

*Rhizophagus irregularis* BEG141 inoculum (200 spores per gram) was thoroughly mixed with vermiculite as the growth substrate. Surface-sterilized soybean seeds (12–16 h chlorine treatment) were sown in the inoculated vermiculite, with two seeds per pot. Plants were maintained in a greenhouse under a 16/8 h light/dark photoperiod at 26 °C. Seven days post-germination, seedlings were thinned to one plant per pot. Non-mycorrhizal control plants were grown in non-inoculated vermiculite under identical conditions. Both treatments received 1/2-strength Hoagland’s low-phosphorus nutrient solution containing 20 μM KH_2_PO_4_ every three days.

Root samples were harvested at the designated AM fungal colonization time points. Mycorrhizal roots were collected at 13, 21, and 26 days post-inoculation and designated as AM13, AM21, and AM26, respectively. Non-mycorrhizal control roots were collected at 21 days and designated as NM21. Each sample group contained three independent biological replicates. Root samples were immediately frozen in liquid nitrogen and stored at −80 °C until use.

For RNA-seq analysis, three independent biological replicates were included for each sample group. The original sequencing output included CK1–CK3, Z_ZQCF1–Z_ZQCF3, Z_ZhQCF1–Z_ZhQCF3, and Z_MQCF1–Z_MQCF3, corresponding to NM21, AM13, AM21, and AM26 in the manuscript, respectively.

### 4.2. RNA-Seq Library Construction, Data Analysis, and qRT-PCR

For RNA-seq, total RNA extraction, RNA integrity assessment, library construction, and sequencing were performed by Nuohe Zhiyuan Biotechnology Co., Ltd. (Beijing, China). RNA integrity was assessed by agarose gel electrophoresis and an Agilent 2100 Bioanalyzer. All RNA samples used for library construction passed the quality-control criteria of the sequencing provider, with RNA integrity number (RIN) values ranging from 6.7 to 9.8. Eukaryotic mRNA was enriched using oligo(dT)-conjugated magnetic beads and fragmented for complementary DNA (cDNA) library construction. First-strand cDNA was synthesized using random hexamer primers, followed by second-strand cDNA synthesis, purification, end repair, A-tailing, adapter ligation, size selection, and PCR enrichment. Library quality was evaluated using Qubit 2.0 fluorometry, insert size assessment was performed with an Agilent 2100 Bioanalyzer, and accurate quantification was performed using quantitative PCR (qPCR) to ensure that the effective library concentration exceeded 2 nM. Quality-validated libraries were pooled at equimolar ratios according to the target sequencing depth and sequenced on the Illumina HiSeq platform.

Raw sequencing reads were filtered to remove adaptor-containing reads, reads with an N proportion greater than 10%, and low-quality reads in which bases with Qphred ≤ 20 accounted for more than 50% of the total read length. Q20, Q30, GC content, and sequencing error rates were calculated to evaluate sequencing quality. The clean reads showed Q20 values of 97.56–98.06%, Q30 values of 92.89–94.12%, and sequencing error rates of 0.03%. The resulting clean reads were aligned to the *Glycine max* reference genome using HISAT with default parameters. Gene-level read counts were generated using HTSeq with the union model. FPKM values were calculated to estimate normalized gene expression levels and were used for expression-level visualization, heatmap analysis, and principal component analysis (PCA). PCA was performed based on log_2_(FPKM + 1)-transformed expression values from all RNA-seq samples to evaluate sample-level clustering patterns and biological replicate reproducibility.

Differential expression analysis was performed using DESeq based on read count data. Differentially expressed genes (DEGs) were identified using a false discovery rate (FDR)-adjusted *p* value (*p*adj) < 0.05 as the significance threshold. No additional absolute log_2_(fold change) cutoff was applied for DEG identification; log_2_(fold change) values were used to indicate the direction and magnitude of expression changes among significant DEGs. Among the significant DEGs, genes with log_2_(fold change) > 0 were classified as upregulated, whereas genes with log_2_(fold change) < 0 were classified as downregulated. GO enrichment analysis of DEGs was performed using GOseq, and KEGG pathway enrichment analysis was performed using KOBAS 2.0. Complete FPKM matrices, raw read count matrices, DEG lists, and GO and KEGG enrichment results are provided in Supplementary Data Sets 1–5.

Quantitative real-time reverse transcription PCR (qRT-PCR) was performed to validate the transcriptome sequencing results and to examine the expression patterns of fatty acid metabolism-related genes in *GmGELP*-targeting hairy roots. For qRT-PCR validation of RNA-seq results, RNA samples were collected from independently grown plants in a separate biological experiment conducted under the same growth and inoculation conditions, with three biological replicates included for each sample group. For qRT-PCR analysis of fatty acid metabolism-related genes in *GmGELP*-targeting hairy roots, three independent biological replicates were also analyzed. RNA samples were quantified using a NanoDrop 2000 spectrophotometer (Thermo Fisher Scientific, Waltham, MA, USA). First-strand cDNA was synthesized from 1 µg of total RNA using HiScript II QRT SuperMix (Vazyme, Nanjing, China). qRT-PCR reactions were conducted with ChamQ Blue Universal SYBR qPCR Master Mix (Vazyme, Nanjing, China) on an Applied Biosystems ViiA 7 Real-Time PCR System. The soybean elongation factor gene *GmEF-1α* was used as the internal reference gene, and relative expression levels were calculated using the 2^−ΔΔCt^ method. Each qRT-PCR assay included three independent biological replicates, and each biological replicate was measured with three technical replicates. The qRT-PCR primers are listed in Supplementary Data Set 8.

### 4.3. Weighted Gene Co-Expression Network Analysis

Weighted gene co-expression network analysis (WGCNA) was performed using TBtools-II (version 2.487) based on FPKM values from the RNA-seq dataset. Prior to network construction, low-expression genes were removed, and genes with a total FPKM value greater than 80 across the analyzed samples were retained, resulting in 16,478 genes. Genes lacking reliable annotation, including unannotated or novel gene models, were further excluded to facilitate downstream functional interpretation, and 15,000 genes were used for co-expression network construction. A soft-thresholding power of 24 was selected, under which the scale-free topology fit index reached R^2^ = 0.89. Modules were identified using a minimum module size of 30, and modules with highly similar expression profiles were merged using a module cut height of 0.25. Module eigengenes were calculated and correlated with sample-group traits corresponding to NM21, AM13, AM21, and AM26. Six representative modules, including brown, green, blue, green-yellow, red, and turquoise, were selected for further analysis based on their sample associations, expression patterns, and functional relevance to AM fungal colonization. Gene lists and filtered co-expression network edge tables for the representative modules are provided in Supplementary Data Sets 6 and 7.

### 4.4. Hairy Root Transformation and Growth Conditions

*Agrobacterium rhizogenes* K599 was used to generate transgenic soybean hairy roots [55,56]. The system was employed to produce CRISPR/Cas9-targeting transgenic hairy roots for phenotypic observation in mycorrhizal symbiosis. GFP-positive transgenic hairy roots were selected under a fluorescence stereomicroscope, and non-transgenic roots were removed. For each composite plant, one robust GFP-positive transgenic hairy root was retained for subsequent inoculation and analysis. Each biological replicate consisted of one independent transgenic hairy root derived from an independent transformation event.

The inoculum of *Rhizophagus irregularis* was thoroughly mixed into the growing medium according to the predetermined inoculum density. Each composite plant carrying one retained GFP-positive hairy root was transferred to the inoculum-containing substrate and cultured in a growth chamber maintained at 26 °C with a 16 h photoperiod (16 h light/8 h dark). Plants were irrigated with 1/2-strength Hoagland low-phosphorus nutrient solution at 3-day intervals. After 28 days of cultivation, plant phenotypes were assessed visually, and root samples were harvested for downstream analyses.

At harvest, three GFP-positive transgenic hairy roots were randomly selected from three independent plants in each *GmGELP*-targeting construct group for genomic DNA extraction and target-site genotyping. For the *GmGELP140/141* dual-targeting construct, both the *GmGELP140* and *GmGELP141* target loci were amplified and genotyped separately in each of the same three selected hairy roots. Regions flanking the sgRNA target sites were amplified by PCR, and the PCR products were cloned into a T-vector. For each target locus in each hairy-root sample, six independent colonies were randomly selected for Sanger sequencing. Samples containing both WT and edited sequences among the six sequenced T-clones were classified as WT/edited mixed genotypes, whereas samples in which only edited sequences were detected among the six sequenced T-clones were classified as edited-only roots. The same genomic DNA samples were used to examine the predicted off-target sites listed in Supplementary Data Set 10 by PCR amplification and Sanger sequencing. Target-site editing patterns, target-site sequencing chromatograms, and predicted off-target-site sequencing chromatograms are shown in Figure S10B–D. Primers used for target-site and predicted off-target-site amplification are listed in Supplementary Data Set 8, whereas the sgRNA target sequences, PAM sequences, on-target scores, target regions, predicted off-target loci, and predicted off-target sequences are provided in Supplementary Data Set 10.

### 4.5. Root Staining and Mycorrhizal Phenotypic Analysis

Mycorrhizal roots were treated with 10% (*w*/*v*) KOH solution for 20–50 min at 95 °C, then washed with 2% HCl. The roots were subsequently stained with 0.5% (*w*/*v*) ink staining solution for 10 min at 95 °C [57].

For wheat germ agglutinin Alexa Fluor^TM^ 488 conjugate (WGA488) staining, AM fungal-colonized soybean roots were fixed in 50% ethanol for 2–4 h. The roots were then cleared in 20% KOH at 75 °C for 48 h, washed thoroughly three times with distilled water, and washed once with phosphate-buffered saline (PBS; pH 7.4). The cleared roots were stained with WGA488 (Thermo Fisher Scientific, Waltham, MA, USA; 0.2–0.4 µg/mL in PBS, pH 7.4) overnight at 4 °C. After staining, roots were washed with PBS and observed under a fluorescence microscope. WGA488-stained images were used to visualize fungal structures and arbuscule morphology, whereas mycorrhizal colonization levels were quantified from ink-stained roots.

For quantification of mycorrhizal colonization, 30 ink-stained root segments were randomly selected and placed on a transparent plate containing 1 cm × 1 cm gridlines. Mycorrhizal structures were quantified using the gridline intersect method under a light microscope [58]. A total of 100 root–gridline intersections were examined for each sample. At each intersection, the presence of internal hyphae, arbuscules, and vesicles was recorded. Total colonization was counted when any fungal structure, including internal hyphae, arbuscules, or vesicles, was present at the intersection. Hyphal abundance, arbuscule abundance, and vesicle abundance were calculated based on the number of intersections containing the corresponding structures.

### 4.6. Plasmid Construction

For gene editing in soybean, target sequences were selected using CRISPR2 (http://crispr.hzau.edu.cn/CRISPR2/). Candidate sgRNAs were evaluated based on their on-target scores, genomic positions, and predicted off-target profiles, and the final sgRNAs were selected for vector construction. The selected sgRNA target sequences, PAM sequences, target regions, on-target scores, and predicted off-target information are provided in Supplementary Data Set 10. Primers used for target-region amplification and predicted off-target-site amplification are listed in Supplementary Data Set 8. For *GmGELP6*, *GmGELP29*, and *GmGELP151*, single-sgRNA constructs were designed. Because *GmGELP140* and *GmGELP141* are tandem duplicate genes with conserved target regions, a dual-targeting CRISPR/Cas9 construct was designed to simultaneously edit both genes. Using pGTR as a template, sgRNA fragments were amplified and ligated into the BbsI-digested intermediate vector pBluescript sk(+)-LjU6 via homologous recombination to form a polycistronic tRNA-gRNA (PTG) structure. After verification by sequencing, the LjU6 promoter and PTG structure were excised from the intermediate vector using KpnI and XbaI restriction enzymes, and then ligated into the pCAMBIA1300 CRISPR/Cas9 binary vector. The pCAMBIA1300-based CRISPR/Cas9 vector contained the Cas9 expression cassette and a GFP marker for screening transgenic hairy roots. The empty-vector control used in this study contained the same CRISPR/Cas9 backbone as the *GmGELP*-editing constructs but lacked *GmGELP*-targeting sgRNA sequences. This control vector was used to generate CK-CRISPR hairy roots.

### 4.7. Statistical Analysis

Morphological and physiological parameters (plant height, fresh weight, and root weight), symbiotic phenotypic parameters, and qRT-PCR data were statistically analyzed using GraphPad Prism 9 (GraphPad Software, San Diego, CA, USA). Data are presented as mean ± standard deviation (SD). For hairy-root experiments, each biological replicate represented one independent soybean composite plant carrying one GFP-positive transgenic hairy root derived from an independent transformation event. Statistical significance was determined using one-way analysis of variance (ANOVA) followed by Dunnett’s multiple comparisons test. Significance levels: * *p* < 0.05; ** *p* < 0.01; *** *p* < 0.001; **** *p* < 0.0001; ns, not significant. Heat maps were generated using TBtools-II (version 2.487).

## Data Availability

RNA sequencing data used in this study are available in the National Center for Biotechnology Information Sequence Read Archive under BioProject accession number PRJNA1348360. This BioProject includes soybean root transcriptome samples from mycorrhizal roots collected at 13, 21, and 26 days post-inoculation, designated as AM13, AM21, and AM26 in the manuscript, as well as non-mycorrhizal control roots collected at the corresponding 21-day time point, designated as NM21. In the original sequencing output, AM13, AM21, AM26, and NM21 correspond to Z_ZQCF, Z_ZhQCF, Z_MQCF, and CK, respectively.

## Supplementary Materials

The following supporting information can be downloaded at: https://www.mdpi.com/article/10.3390/ijms27156605/s1.
