# Supplementary material for: Transcriptomic Profiling Identifies Symbiosis-Induced GDSL Lipase Genes Associated with Soybean–Arbuscular Mycorrhizal Symbiosis
Source: Int J Mol Sci. 2026 Jul 24;27(15):6605. doi: 10.3390/ijms27156605 (PMC13466371; doi:10.3390/ijms27156605)
Supplement: Supplementary file 1 [file ijms-27-06605-s001.zip › Supplementary Figures and Tables.pdf]

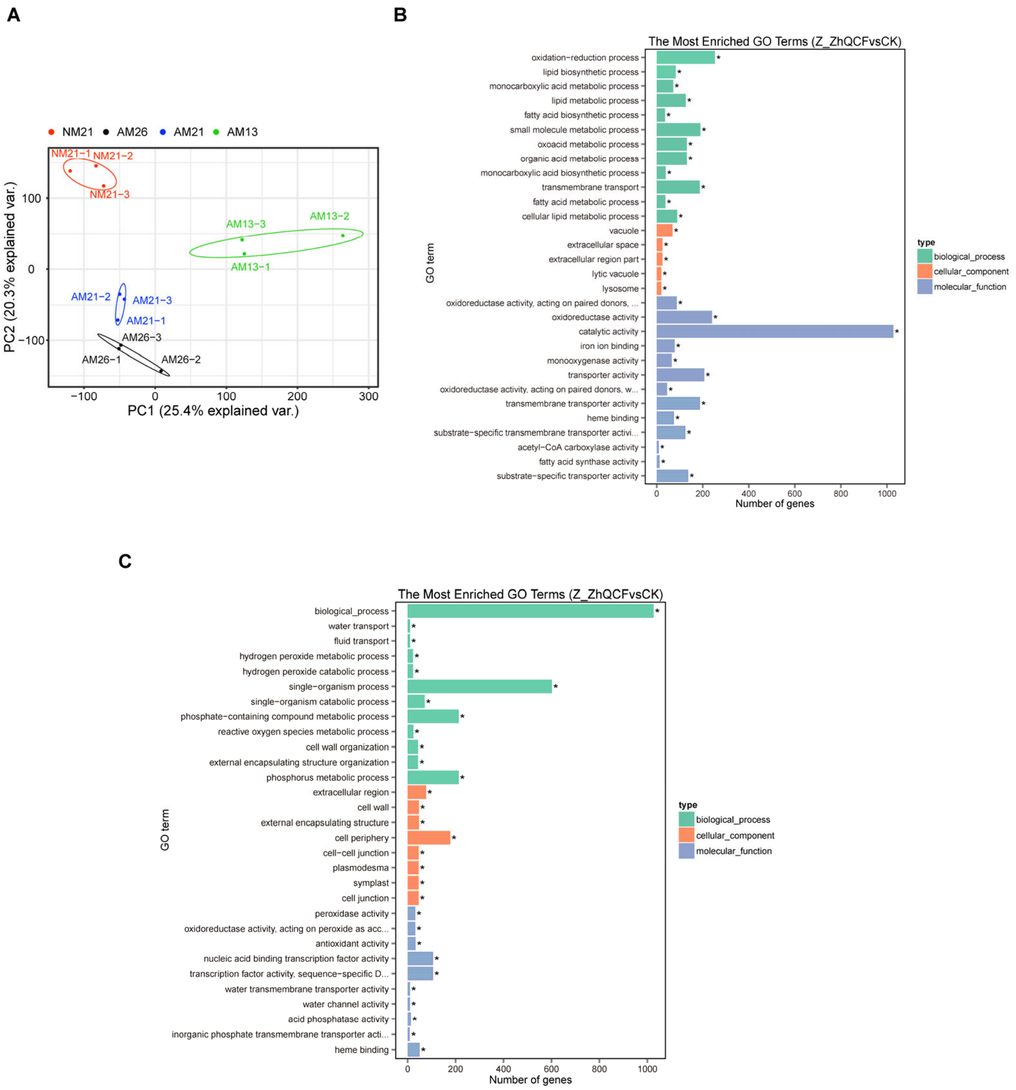
A
B
C
NM21
AM26
AM21
AM13
NM21−1
NM21−2
NM21−3
AM13−3
AM13−2
AM13−1
AM21−2
AM21−3
AM21−1
AM26−3
AM26−1
AM26−2
PC2 (20.3% explained var.)
PC1 (25.4% explained var.)
100
0
−100
−100
0
100
200
300
The Most Enriched GO Terms (Z_ZhQCFvsCK)
oxidation−reduction process
lipid biosynthetic process
monocarboxylic acid metabolic process
lipid metabolic process
fatty acid biosynthetic process
small molecule metabolic process
oxoacid metabolic process
organic acid metabolic process
monocarboxylic acid biosynthetic process
transmembrane transport
fatty acid metabolic process
cellular lipid metabolic process
vacuole
extracellular space
extracellular region part
lytic vacuole
lysosome
oxidoreductase activity, acting on paired donors, ...
oxidoreductase activity
catalytic activity
iron ion binding
monooxygenase activity
transporter activity
oxidoreductase activity, acting on paired donors, w...
transmembrane transporter activity
heme binding
substrate−specific transmembrane transporter activi...
acetyl−CoA carboxylase activity
fatty acid synthase activity
substrate−specific transporter activity
GO term
Number of genes
0
200
400
600
800
1000
type
biological_process
cellular_component
molecular_function
The Most Enriched GO Terms (Z_ZhQCFvsCK)
biological_process
water transport
fluid transport
hydrogen peroxide metabolic process
hydrogen peroxide catabolic process
single−organism process
single−organism catabolic process
phosphate−containing compound metabolic process
reactive oxygen species metabolic process
cell wall organization
external encapsulating structure organization
phosphorus metabolic process
extracellular region
cell wall
external encapsulating structure
cell periphery
cell−cell junction
plasmodesma
symplast
cell junction
peroxidase activity
oxidoreductase activity, acting on peroxide as acc...
antioxidant activity
nucleic acid binding transcription factor activity
transcription factor activity, sequence−specific D...
water transmembrane transporter activity
water channel activity
acid phosphatase activity
inorganic phosphate transmembrane transporter acti...
heme binding
GO term
Number of genes
0
200
400
600
800
1000
type
biological_process
cellular_component
molecular_function

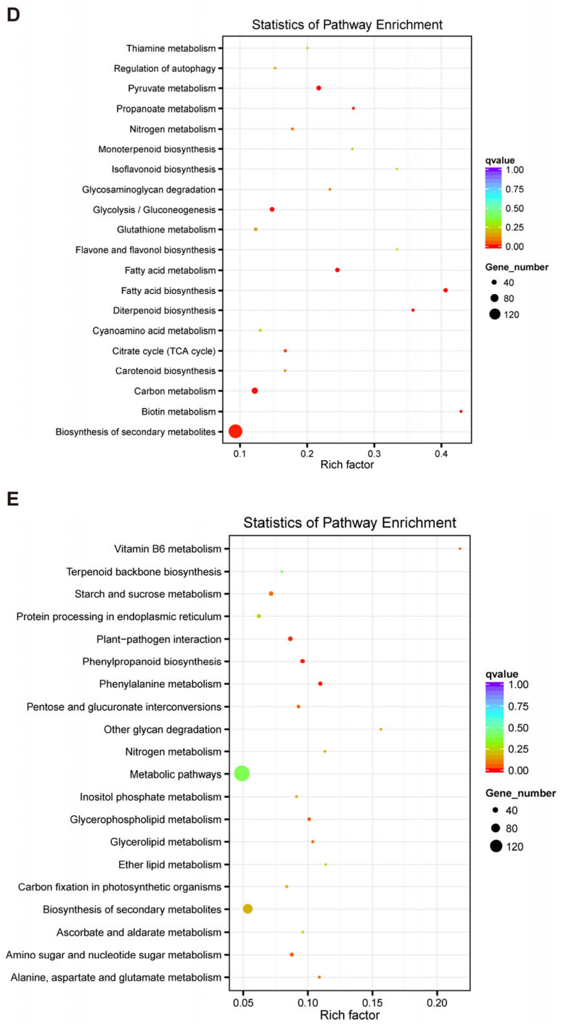

Fig. S1. PCA analysis of RNA-seq samples and GO/KEGG enrichment analysis of differentially expressed genes (DEGs) in the AM21 vs. NM21 comparison.

(A) Principal component analysis (PCA) of RNA-seq samples based on $\log_2$(FPKM + 1) values. Each point represents one biological replicate. NM21 represents 21-day non-mycorrhizal soybean roots, whereas AM13, AM21, and AM26 represent mycorrhizal soybean roots collected at 13, 21, and 26 days after inoculation, respectively. Ellipses indicate sample grouping.

(B) GO enrichment analysis of upregulated DEGs in the AM21 vs. NM21 comparison.

(C) GO enrichment analysis of downregulated DEGs in the AM21 vs. NM21 comparison. For GO enrichment plots, the x-axis indicates the number of DEGs assigned to each GO term, and the y-axis shows the enriched GO terms. Different colors distinguish biological process, cellular component, and molecular function categories. Asterisks (*) indicate significantly enriched GO

terms (adjusted *P* value [padj] < 0.05).

(D) KEGG enrichment analysis of upregulated DEGs in the AM21 vs. NM21 comparison.

(E) KEGG enrichment analysis of downregulated DEGs in the AM21 vs. NM21 comparison.

For KEGG enrichment plots, the x-axis indicates the rich factor, dot size represents the number of genes enriched in each pathway, and dot color indicates the enrichment significance.

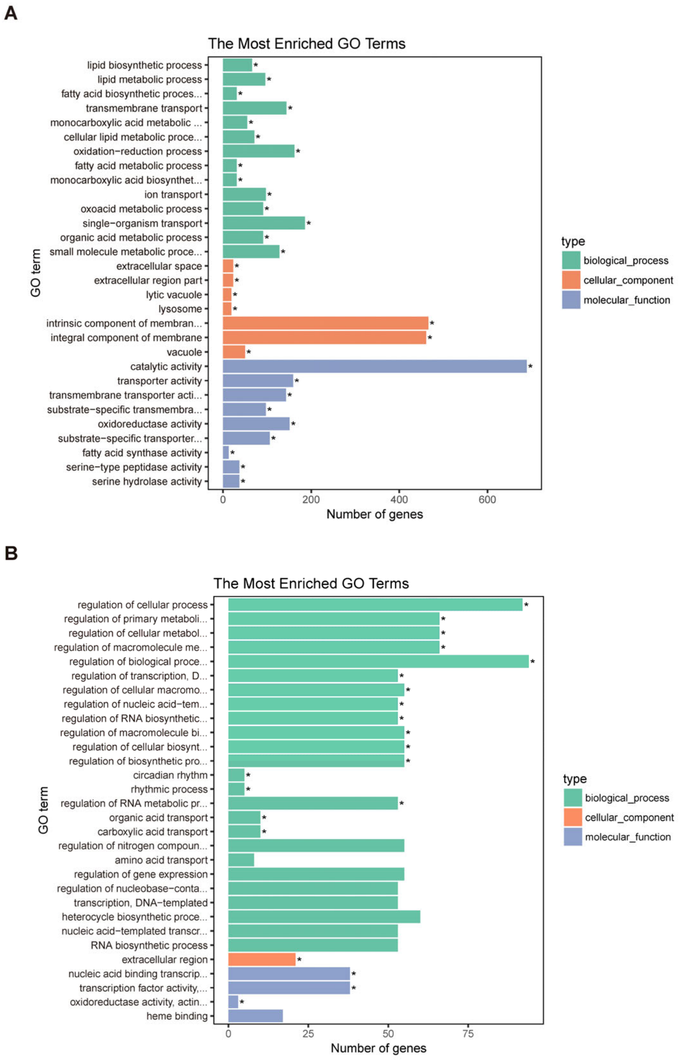
A
The Most Enriched GO Terms
GO term
lipid biosynthetic process
lipid metabolic process
fatty acid biosynthetic proces...
transmembrane transport
monocarboxylic acid metabolic ...
cellular lipid metabolic proce...
oxidation−reduction process
fatty acid metabolic process
monocarboxylic acid biosynthet...
ion transport
oxoacid metabolic process
single−organism transport
organic acid metabolic process
small molecule metabolic proce...
extracellular space
extracellular region part
lytic vacuole
lysosome
intrinsic component of membran...
integral component of membrane
vacuole
catalytic activity
transporter activity
transmembrane transporter acti...
substrate−specific transmembra...
oxidoreductase activity
substrate−specific transporter...
fatty acid synthase activity
serine−type peptidase activity
serine hydrolase activity
0
200
400
600
Number of genes
type
biological_process
cellular_component
molecular_function
B
The Most Enriched GO Terms
GO term
regulation of cellular process
regulation of primary metaboli...
regulation of cellular metabol...
regulation of macromolecule me...
regulation of biological proce...
regulation of transcription, D...
regulation of cellular macromo...
regulation of nucleic acid−tem...
regulation of RNA biosynthetic...
regulation of macromolecule bi...
regulation of cellular biosynt...
regulation of biosynthetic pro...
circadian rhythm
rhythmic process
regulation of RNA metabolic pr...
organic acid transport
carboxylic acid transport
regulation of nitrogen compoun...
amino acid transport
regulation of gene expression
regulation of nucleobase−conta...
transcription, DNA−templated
heterocycle biosynthetic proce...
nucleic acid−templated transcr...
RNA biosynthetic process
extracellular region
nucleic acid binding transcrip...
transcription factor activity,...
oxidoreductase activity, actin...
heme binding
0
25
50
75
Number of genes
type
biological_process
cellular_component
molecular_function

C

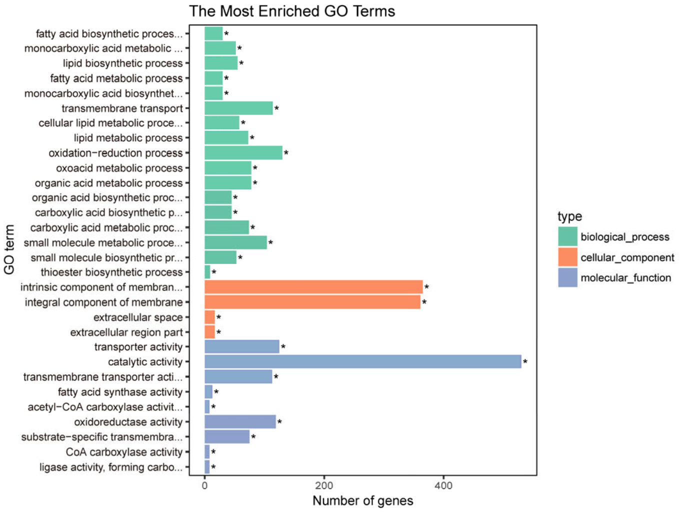
The Most Enriched GO Terms
fatty acid biosynthetic proces...
monocarboxylic acid metabolic ...
lipid biosynthetic process
fatty acid metabolic process
monocarboxylic acid biosynthet...
transmembrane transport
cellular lipid metabolic proce...
lipid metabolic process
oxidation−reduction process
oxoacid metabolic process
organic acid metabolic process
organic acid biosynthetic proc...
carboxylic acid biosynthetic p...
carboxylic acid metabolic proc...
small molecule metabolic proce...
small molecule biosynthetic pr...
thioester biosynthetic process
intrinsic component of membran...
integral component of membrane
extracellular space
extracellular region part
transporter activity
catalytic activity
transmembrane transporter acti...
fatty acid synthase activity
acetyl−CoA carboxylase activit...
oxidoreductase activity
substrate−specific transmembra...
CoA carboxylase activity
ligase activity, forming carbo...
GO term
0
200
400
Number of genes
type
biological_process
cellular_component
molecular_function

D

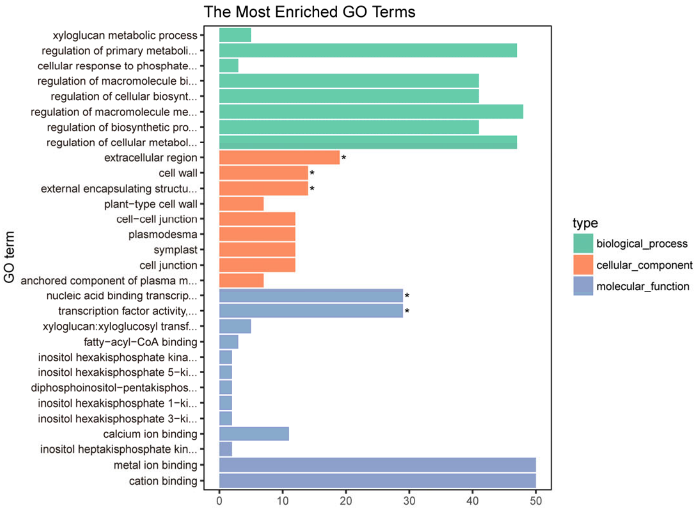
The Most Enriched GO Terms
xyloglucan metabolic process
regulation of primary metaboli...
cellular response to phosphate...
regulation of macromolecule bi...
regulation of cellular biosynt...
regulation of macromolecule me...
regulation of biosynthetic pro...
regulation of cellular metabol...
extracellular region
cell wall
external encapsulating structu...
plant−type cell wall
cell−cell junction
plasmodesma
symplast
cell junction
anchored component of plasma m...
nucleic acid binding transcrip...
transcription factor activity,...
xyloglucan:xyloglucosyl transf...
fatty−acyl−CoA binding
inositol hexakisphosphate kina...
inositol hexakisphosphate 5−ki...
diphosphoinositol−pentakisphos...
inositol hexakisphosphate 1−ki...
inositol hexakisphosphate 3−ki...
calcium ion binding
inositol heptakisphosphate kin...
metal ion binding
cation binding
GO term
0
10
20
30
40
50
type
biological_process
cellular_component
molecular_function

E

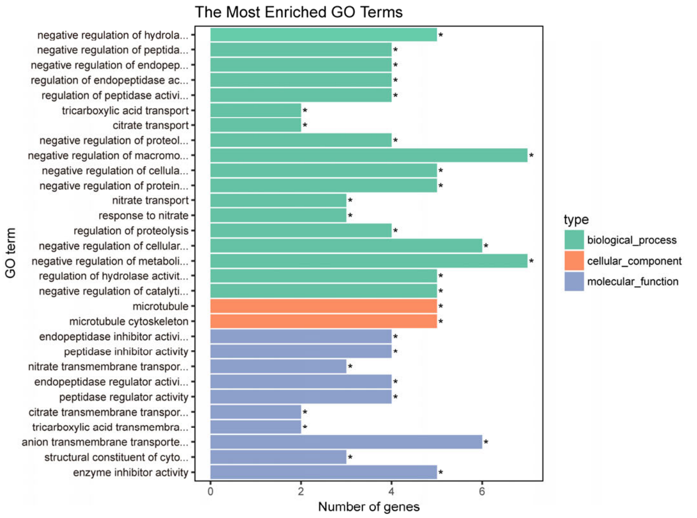

F

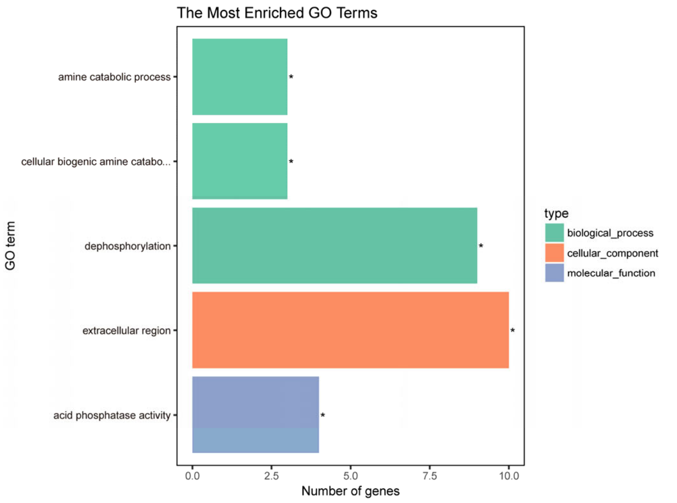

Fig. S2. GO enrichment analysis of symbiotic differentially expressed genes (DEGs).

(A–B) GO enrichment analysis of upregulated and downregulated symbiotic DEGs in the AM13 vs. AM21 comparison.

(C–D) GO enrichment analysis of upregulated and downregulated symbiotic DEGs in the AM13 vs. AM26 comparison.

(E–F) GO enrichment analysis of upregulated and downregulated symbiotic DEGs in the AM21 vs. AM26 comparison.

For GO enrichment plots, the x-axis indicates the number of DEGs assigned to each GO term, and the y-axis shows the enriched GO terms. Different colors distinguish biological process, cellular component, and molecular function categories. Asterisks (*) indicate significantly enriched GO terms (adjusted *P* value [padj] < 0.05).

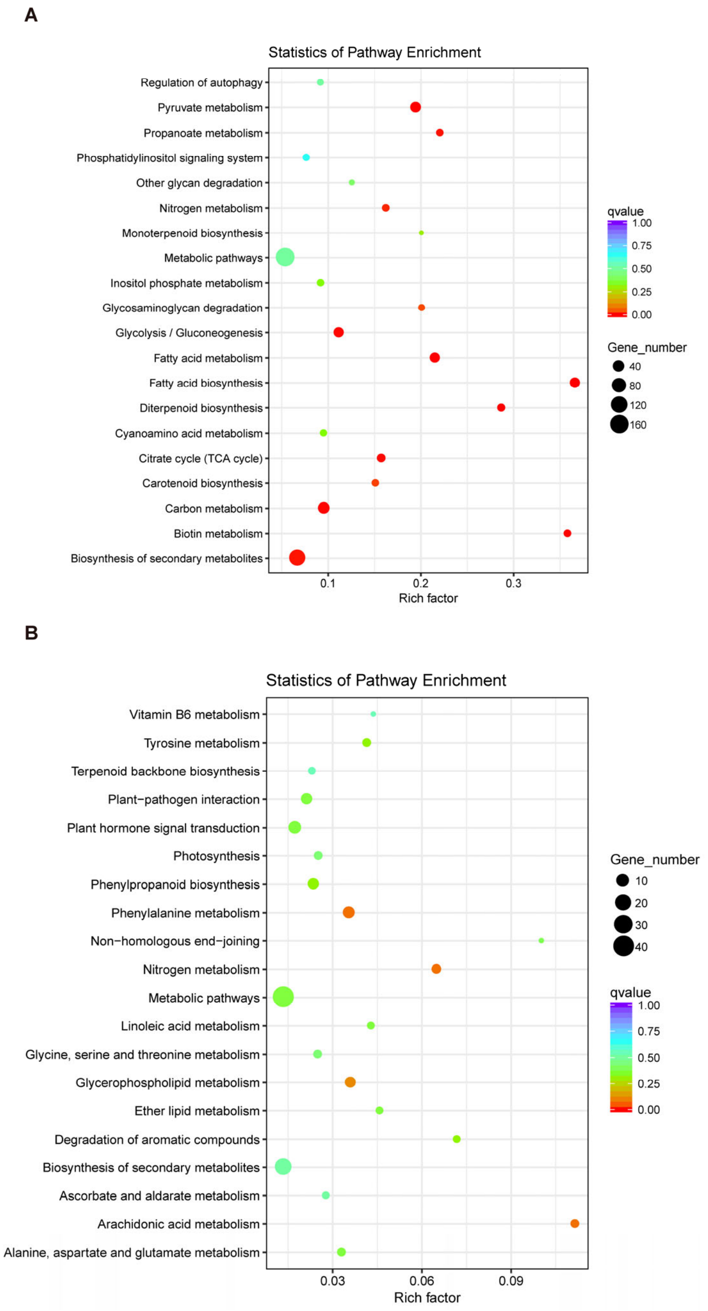
A
Statistics of Pathway Enrichment
Regulation of autophagy
Pyruvate metabolism
Propanoate metabolism
Phosphatidylinositol signaling system
Other glycan degradation
Nitrogen metabolism
Monoterpenoid biosynthesis
Metabolic pathways
Inositol phosphate metabolism
Glycosaminoglycan degradation
Glycolysis / Gluconeogenesis
Fatty acid metabolism
Fatty acid biosynthesis
Diterpenoid biosynthesis
Cyanoamino acid metabolism
Citrate cycle (TCA cycle)
Carotenoid biosynthesis
Carbon metabolism
Biotin metabolism
Biosynthesis of secondary metabolites
0.1
0.2
0.3
Rich factor
qvalue
1.00
0.75
0.50
0.25
0.00
Gene_number
40
80
120
160
B
Statistics of Pathway Enrichment
Vitamin B6 metabolism
Tyrosine metabolism
Terpenoid backbone biosynthesis
Plant−pathogen interaction
Plant hormone signal transduction
Photosynthesis
Phenylpropanoid biosynthesis
Phenylalanine metabolism
Non−homologous end−joining
Nitrogen metabolism
Metabolic pathways
Linoleic acid metabolism
Glycine, serine and threonine metabolism
Glycerophospholipid metabolism
Ether lipid metabolism
Degradation of aromatic compounds
Biosynthesis of secondary metabolites
Ascorbate and aldarate metabolism
Arachidonic acid metabolism
Alanine, aspartate and glutamate metabolism
0.03
0.06
0.09
Rich factor
Gene_number
10
20
30
40
qvalue
1.00
0.75
0.50
0.25
0.00

C

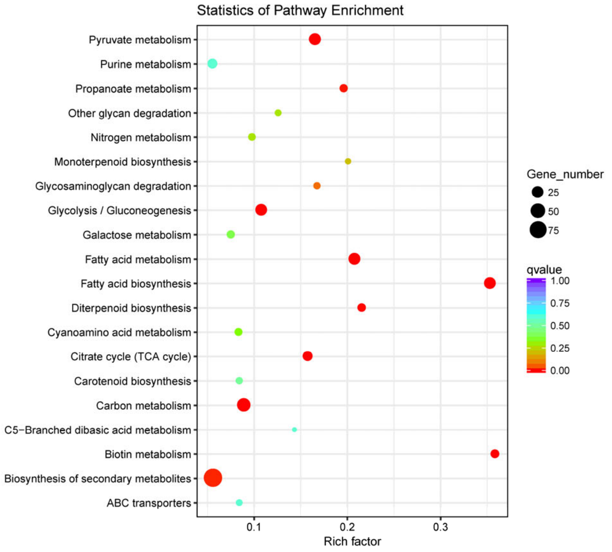

Statistics of Pathway Enrichment
Pyruvate metabolism
Purine metabolism
Propanoate metabolism
Other glycan degradation
Nitrogen metabolism
Monoterpenoid biosynthesis
Glycosaminoglycan degradation
Glycolysis / Gluconeogenesis
Galactose metabolism
Fatty acid metabolism
Fatty acid biosynthesis
Diterpenoid biosynthesis
Cyanoamino acid metabolism
Citrate cycle (TCA cycle)
Carotenoid biosynthesis
Carbon metabolism
C5−Branched dibasic acid metabolism
Biotin metabolism
Biosynthesis of secondary metabolites
ABC transporters
0.1
0.2
0.3
Rich factor
Gene_number
25
50
75
qvalue
1.00
0.75
0.50
0.25
0.00

D

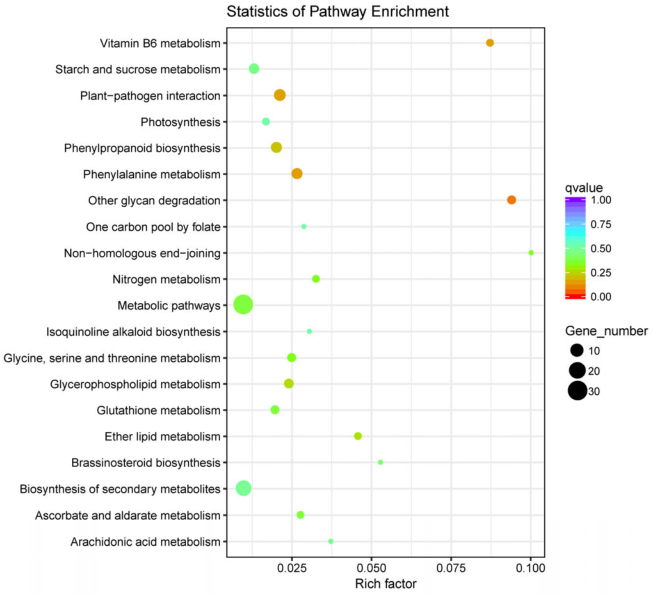

Statistics of Pathway Enrichment
Vitamin B6 metabolism
Starch and sucrose metabolism
Plant−pathogen interaction
Photosynthesis
Phenylpropanoid biosynthesis
Phenylalanine metabolism
Other glycan degradation
One carbon pool by folate
Non−homologous end−joining
Nitrogen metabolism
Metabolic pathways
Isoquinoline alkaloid biosynthesis
Glycine, serine and threonine metabolism
Glycerophospholipid metabolism
Glutathione metabolism
Ether lipid metabolism
Brassinosteroid biosynthesis
Biosynthesis of secondary metabolites
Ascorbate and aldarate metabolism
Arachidonic acid metabolism
0.025
0.050
0.075
0.100
Rich factor
qvalue
1.00
0.75
0.50
0.25
0.00
Gene_number
10
20
30

E

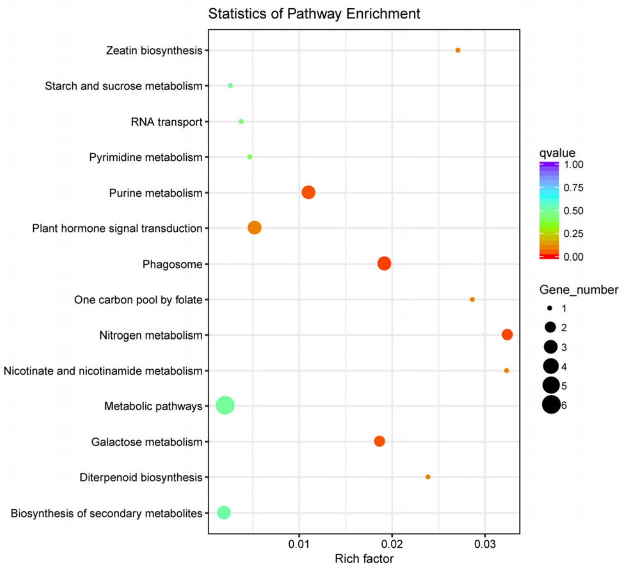

F

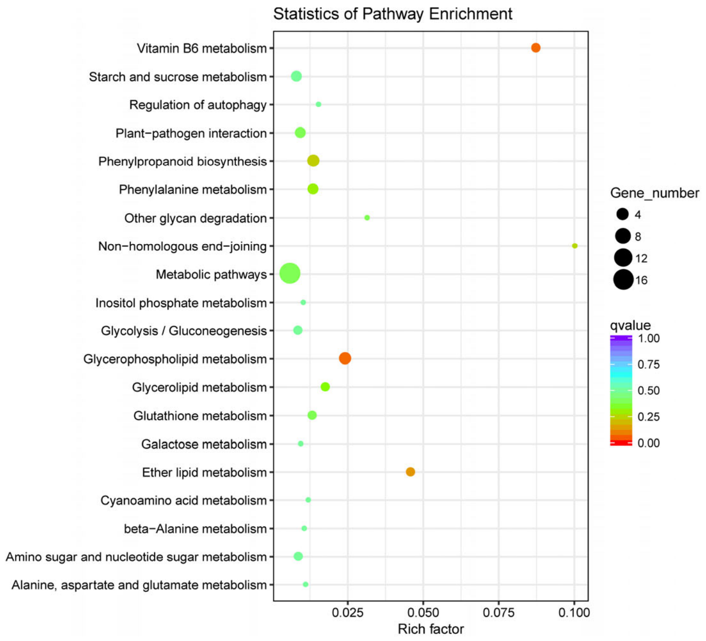

Fig. S3. KEGG enrichment analysis of symbiotic differentially expressed genes (DEGs).

(A–B) KEGG enrichment analysis of upregulated and downregulated symbiotic DEGs in the AM13 vs. AM21 comparison.

(C–D) KEGG enrichment analysis of upregulated and downregulated symbiotic DEGs in the AM13 vs. AM26 comparison.

(E–F) KEGG enrichment analysis of upregulated and downregulated symbiotic DEGs in the AM21 vs. AM26 comparison.

The x-axis indicates the rich factor of KEGG pathways, dot size represents the number of genes

enriched in each pathway, and dot color indicates the significance of enrichment.

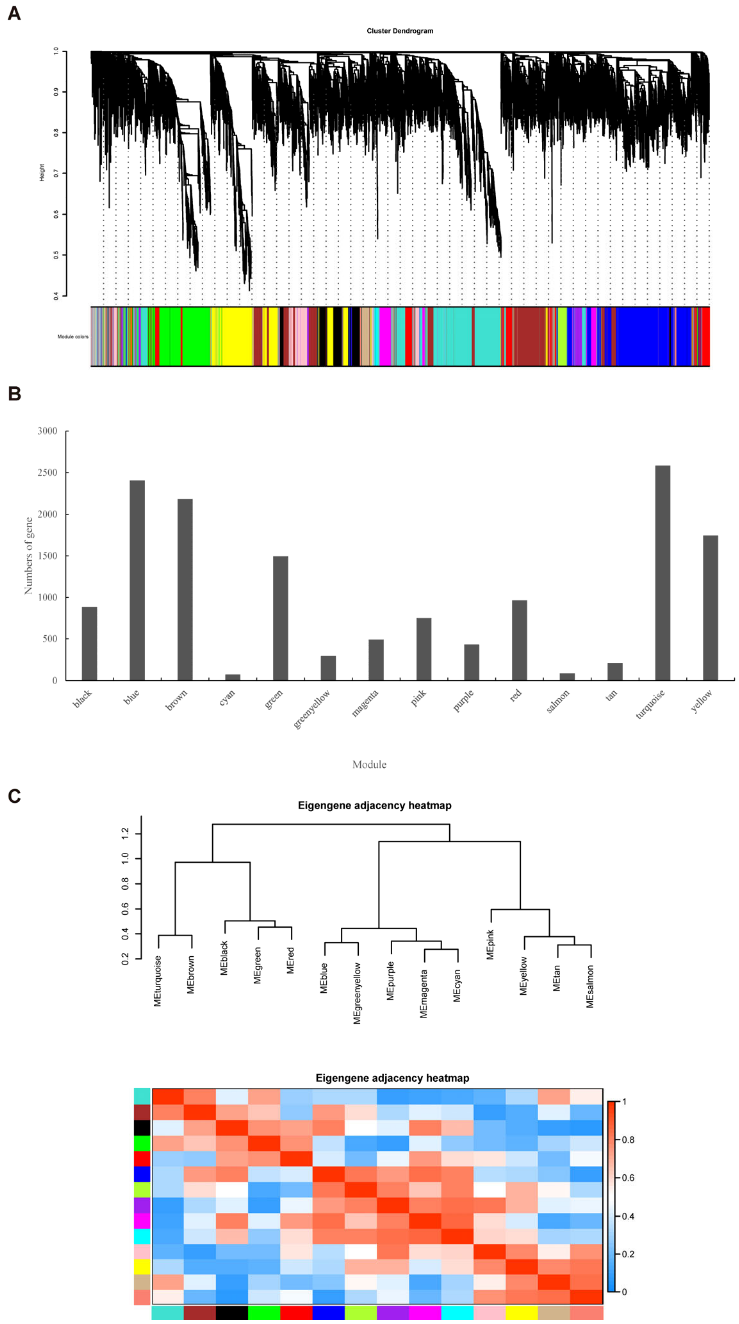

Fig. S4. WGCNA module clustering and module statistics.

(A) Cluster dendrogram of genes used for WGCNA module detection. Each color represents one co-expression module.

(B) Number of genes assigned to each WGCNA module.

(C) Module eigengene clustering and eigengene adjacency heatmap.

A soft-thresholding power value of 24 was selected to construct the scale-free co-expression network, resulting in 14 co-expression modules, each represented by a different color.

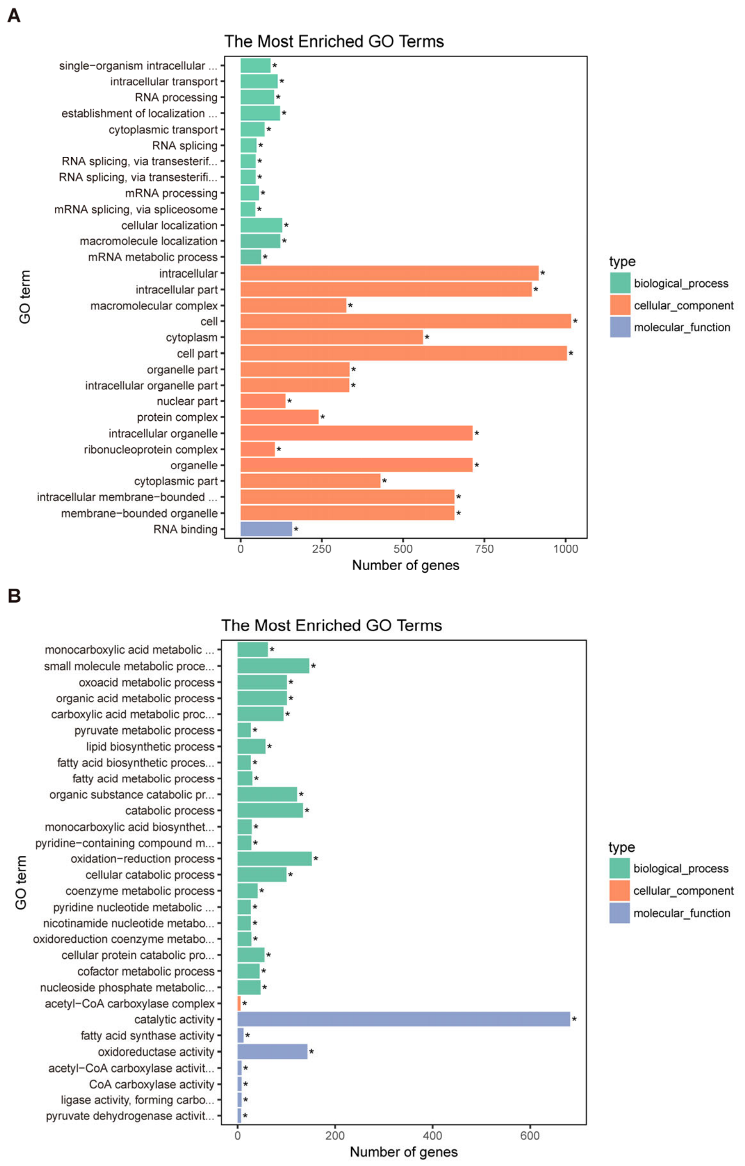
A
The Most Enriched GO Terms
single−organism intracellular ...
intracellular transport
RNA processing
establishment of localization ...
cytoplasmic transport
RNA splicing
RNA splicing, via transesterif...
RNA splicing, via transesterifi...
mRNA processing
mRNA splicing, via spliceosome
cellular localization
macromolecule localization
mRNA metabolic process
intracellular
intracellular part
macromolecular complex
cell
cytoplasm
cell part
organelle part
intracellular organelle part
nuclear part
protein complex
intracellular organelle
ribonucleoprotein complex
organelle
cytoplasmic part
intracellular membrane−bounded ...
membrane−bounded organelle
RNA binding
GO term
0
250
500
750
1000
Number of genes
type
biological_process
cellular_component
molecular_function
B
The Most Enriched GO Terms
monocarboxylic acid metabolic ...
small molecule metabolic proce...
oxoacid metabolic process
organic acid metabolic process
carboxylic acid metabolic proc...
pyruvate metabolic process
lipid biosynthetic process
fatty acid biosynthetic proces...
fatty acid metabolic process
organic substance catabolic pr...
catabolic process
monocarboxylic acid biosynthet...
pyridine−containing compound m...
oxidation−reduction process
cellular catabolic process
coenzyme metabolic process
pyridine nucleotide metabolic ...
nicotinamide nucleotide metabo...
oxidoreduction coenzyme metabo...
cellular protein catabolic pro...
cofactor metabolic process
nucleoside phosphate metabolic...
acetyl−CoA carboxylase complex
catalytic activity
fatty acid synthase activity
oxidoreductase activity
acetyl−CoA carboxylase activit...
CoA carboxylase activity
ligase activity, forming carbo...
pyruvate dehydrogenase activit...
GO term
0
200
400
600
Number of genes
type
biological_process
cellular_component
molecular_function

C

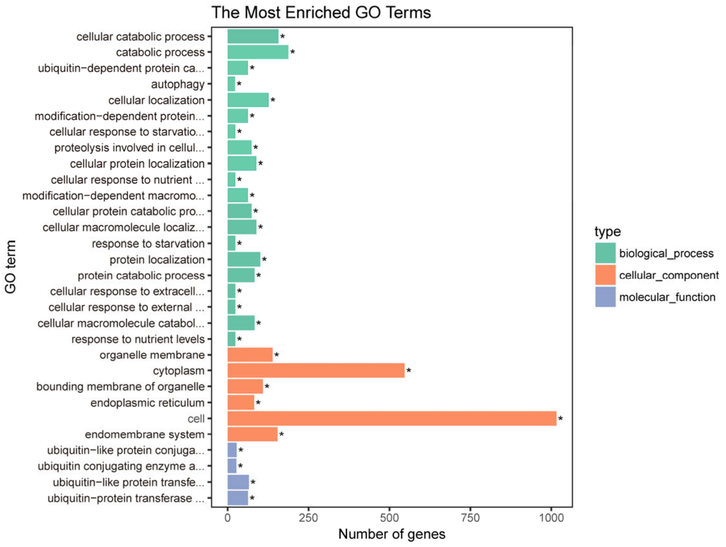
The Most Enriched GO Terms
cellular catabolic process
catabolic process
ubiquitin−dependent protein ca...
autophagy
cellular localization
modification−dependent protein...
cellular response to starvatio...
proteolysis involved in cellul...
cellular protein localization
cellular response to nutrient ...
modification−dependent macromo...
cellular protein catabolic pro...
cellular macromolecule localiz...
response to starvation
protein localization
protein catabolic process
cellular response to extracell...
cellular response to external ...
cellular macromolecule catabol...
response to nutrient levels
organelle membrane
cytoplasm
bounding membrane of organelle
endoplasmic reticulum
cell
endomembrane system
ubiquitin−like protein conjuga...
ubiquitin conjugating enzyme a...
ubiquitin−like protein transfe...
ubiquitin−protein transferase ...
GO term
0
250
500
750
1000
Number of genes
type
biological_process
cellular_component
molecular_function

D

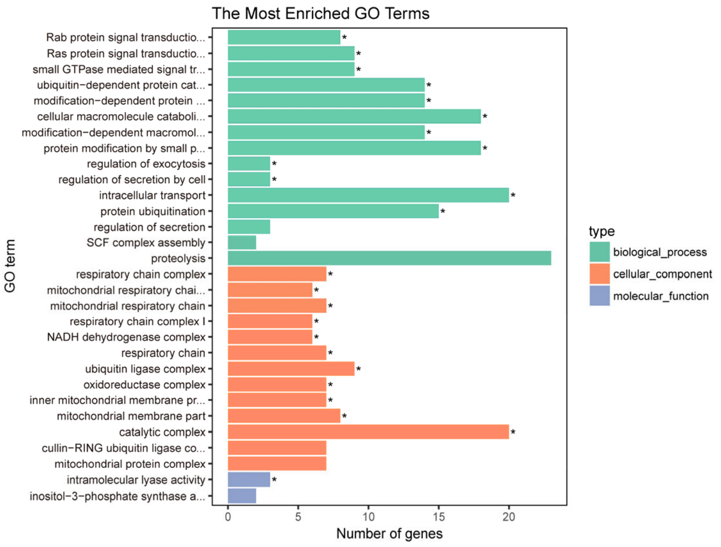
The Most Enriched GO Terms
Rab protein signal transductio...
Ras protein signal transductio...
small GTPase mediated signal tr...
ubiquitin−dependent protein cat...
modification−dependent protein ...
cellular macromolecule cataboli...
modification−dependent macromol...
protein modification by small p...
regulation of exocytosis
regulation of secretion by cell
intracellular transport
protein ubiquitination
regulation of secretion
SCF complex assembly
proteolysis
respiratory chain complex
mitochondrial respiratory chai...
mitochondrial respiratory chain
respiratory chain complex I
NADH dehydrogenase complex
respiratory chain
ubiquitin ligase complex
oxidoreductase complex
inner mitochondrial membrane pr...
mitochondrial membrane part
catalytic complex
cullin−RING ubiquitin ligase co...
mitochondrial protein complex
intramolecular lyase activity
inositol−3−phosphate synthase a...
GO term
0
5
10
15
20
Number of genes
type
biological_process
cellular_component
molecular_function

E

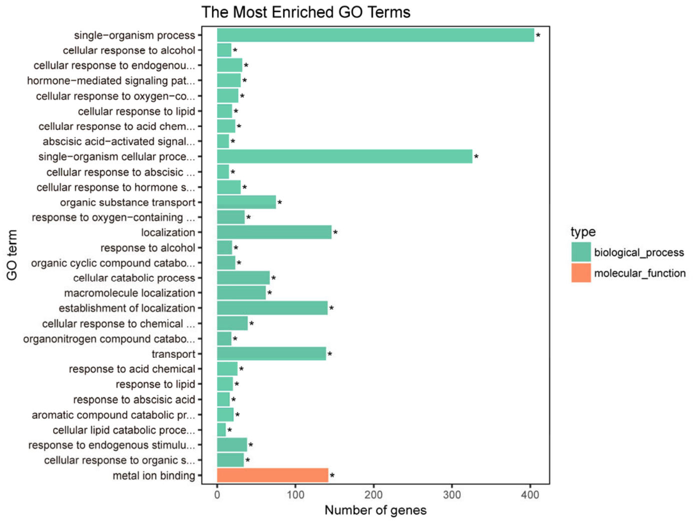

F

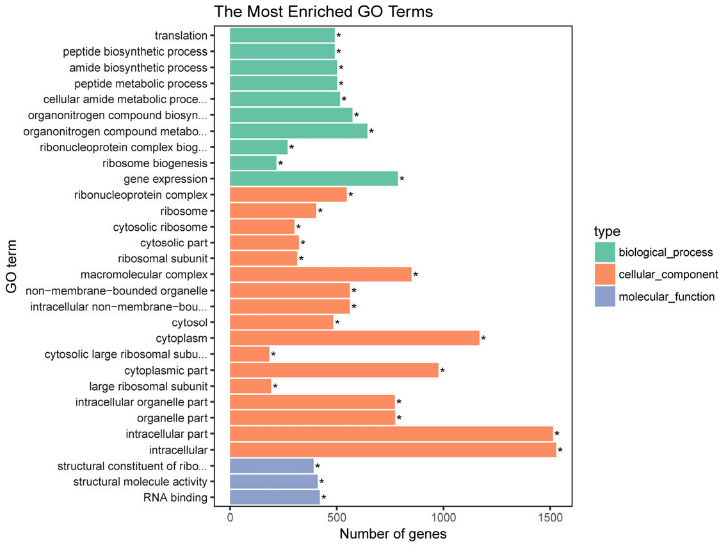

Fig. S5. GO enrichment analysis of genes in symbiosis-associated WGCNA modules.

(A–F) GO enrichment analysis of genes in the brown, green, blue, greenyellow, red, and turquoise modules, respectively.

For GO enrichment plots, the x-axis indicates the number of genes assigned to each GO term, and the y-axis shows the enriched GO terms. Different colors distinguish biological process, cellular component, and molecular function categories. Asterisks (*) indicate significantly enriched GO terms (adjusted *P* value [padj] < 0.05).

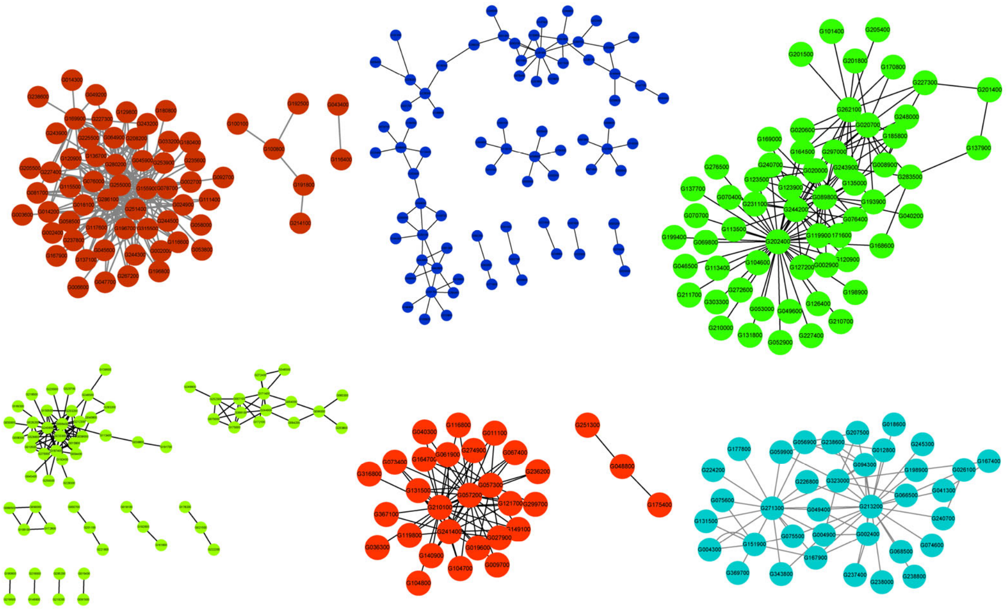

Fig. S6. Co-expression networks of symbiosis-associated WGCNA modules.

Network representation of genes within six symbiosis-associated WGCNA modules. Dots represent nodes, corresponding to genes in the network, and lines represent co-expression relationships between genes. Genes located near the center of each network have higher connectivity, indicating a larger number of connections with other genes in the same module.

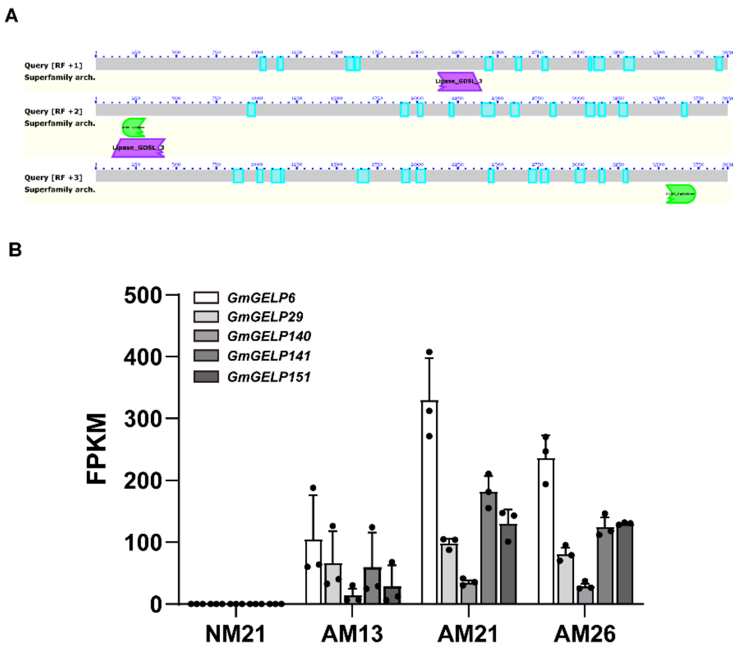

Fig. S7. Domain information and expression patterns of candidate *GmGELP* genes.

(A) Representative conserved domain architecture of a GDSL lipase family protein, using *GmGELP6* as an example. Predicted conserved domains associated with the GDSL/SGNH lipase family are shown.

(B) FPKM expression levels of five candidate *GmGELP* genes, *GmGELP6*, *GmGELP29*, *GmGELP140*, *GmGELP141*, and *GmGELP151*, in NM21, AM13, AM21, and AM26 samples. Data are presented as mean ± SD, and each dot represents one biological replicate.

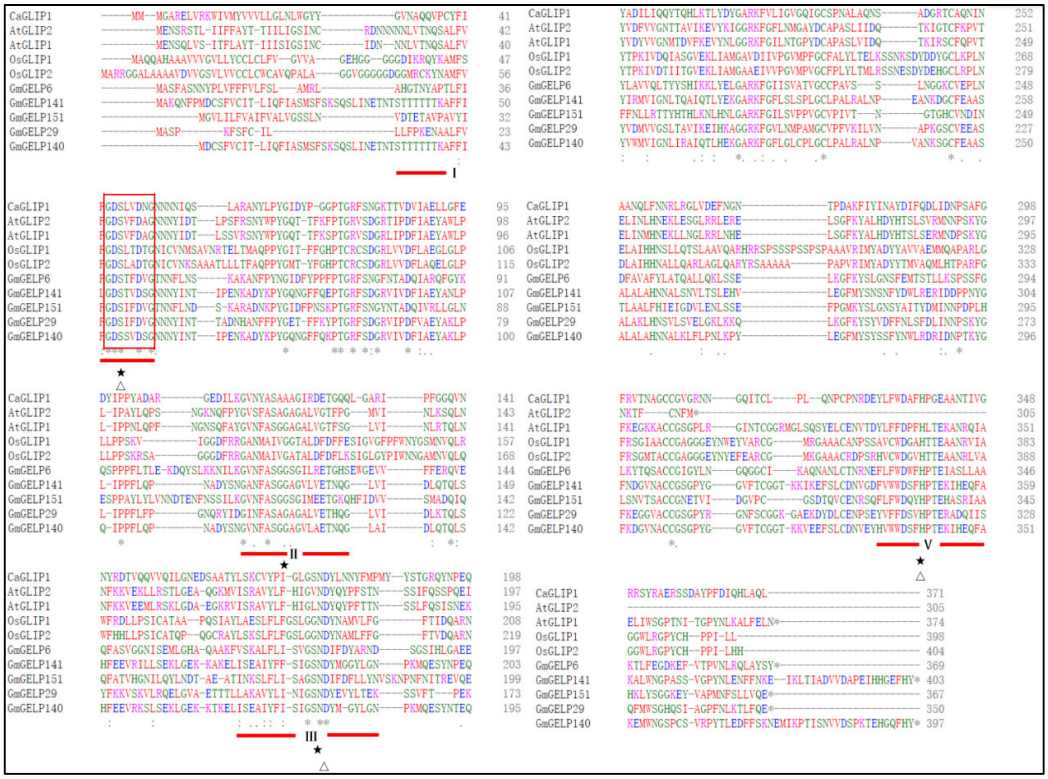

Fig. S8. Multiple sequence alignment of GmGELP6, GmGELP29, GmGELP140, GmGELP141, and GmGELP151 amino acid sequences.

Red lines indicate conserved amino acid regions I, II, III, and V. The red box highlights the conserved GDSL-specific GDSxxDxG motif. Stars indicate fully conserved catalytic residues, while triangles indicate the catalytic triad. Different colors represent amino acids with different biochemical properties. Fully conserved amino acids are indicated by (*), similar amino acids by (:), and weakly similar amino acids by (.), with blanks indicating non-conserved positions.

A

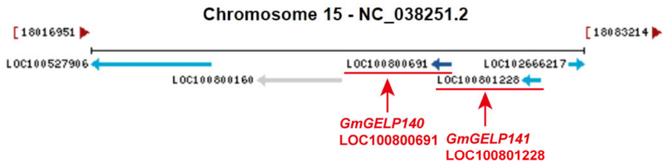

B

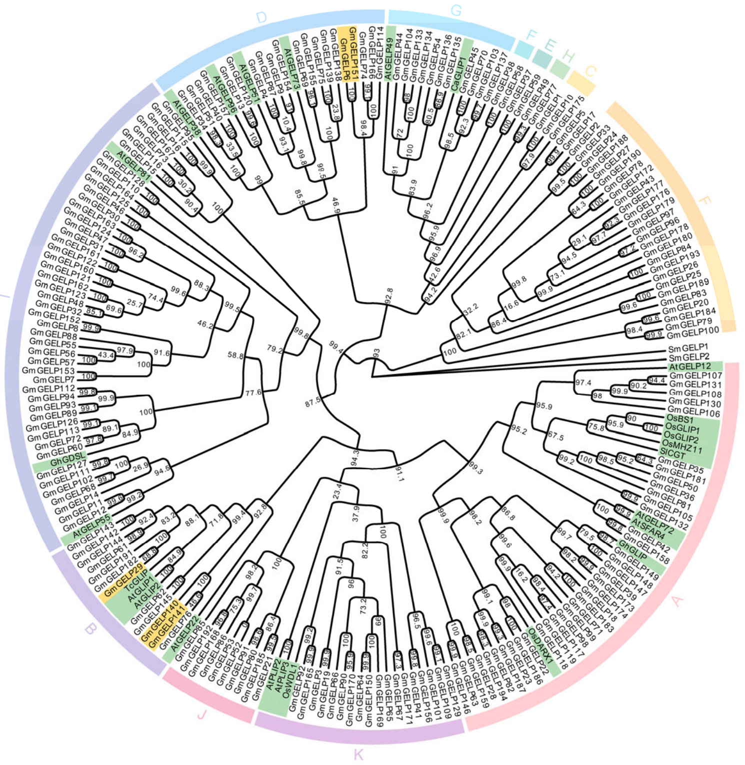

Fig. S9. Chromosomal location of *GmGELP140* and *GmGELP141* and phylogenetic tree of the GELP family.

(A) Chromosomal locations of *GmGELP140* and *GmGELP141* on chromosome 15. Line segments represent chromosomal regions, arrows indicate gene positions and transcriptional directions, and red arrows mark *GmGELP140* and *GmGELP141*.

(B) Phylogenetic tree of GELP family proteins. Different colored letters represent different GELP subgroups (A–K). Proteins with yellow backgrounds are the candidate *GmGELPs* identified in this study, proteins with green backgrounds are previously studied GELPs, and genes without background color are other GELPs.

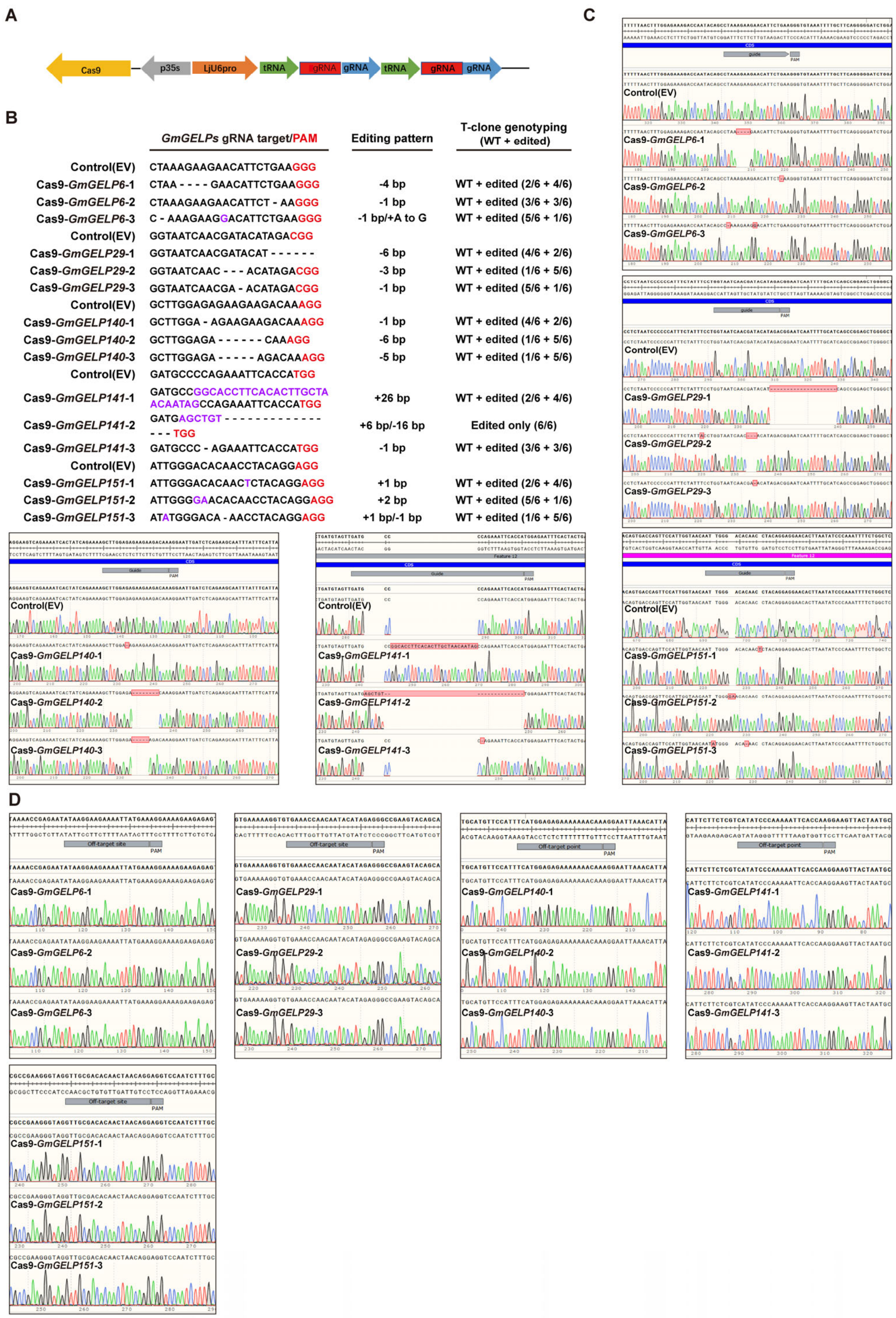

Fig. S10. CRISPR/Cas9-mediated editing of *GmGELPs* in soybean transgenic hairy roots.

(A) Schematic diagrams of the CRISPR/Cas9 vectors used for *GmGELP* editing. The dual-targeting construct for *GmGELP140/141* contained two gRNA expression cassettes

to separately target the tandem duplicated genes *GmGELP140* and *GmGELP141*, whereas the constructs targeting *GmGELP6*, *GmGELP29*, or *GmGELP151* contained a single gRNA expression cassette.

(B) Target-site genotyping and edited sequence patterns of *GmGELP*-targeting soybean hairy roots. The sgRNA target sequences and PAM sequences are shown, with PAM sequences highlighted in red. Hyphens indicate deleted nucleotides, and purple letters indicate inserted or substituted nucleotides. For each hairy-root sample, the PCR product spanning the sgRNA target site was cloned into a T-vector, and six independent T-clones were randomly selected for Sanger sequencing for each target locus. For the *GmGELP140/141* dual-targeting construct, the *GmGELP140* and *GmGELP141* target loci were analyzed separately in each of the same three hairy roots. Mutations at both loci were detected in all three genotyped roots. In the T-clone genotyping column, WT + edited indicates that both WT and edited sequences were detected among the six sequenced T-clones, whereas edited only indicates that all six sequenced T-clones carried edited sequences without detectable WT sequence. The first and second numbers indicate the numbers of WT and edited T-clones, respectively. For each edited hairy-root sample, only the edited sequence pattern is shown; WT T-clone sequences were included in the genotyping counts but are not displayed because they were identical to the control/reference sequence.

(C) Sanger sequencing chromatograms of edited T-clones showing target-site mutations in *GmGELP*-targeting hairy roots. WT T-clone chromatograms from WT + edited samples are not shown because their sequences matched the control/reference sequence.

(D) Sanger sequencing chromatograms of the tested predicted off-target sites in the same genotyped *GmGELP*-targeting hairy roots. No sequence alteration was detected at the examined predicted off-target sites.

Table S1 Information on RNA-seq samples.

Sample name	Organ	Abbreviation	Biological repeat
NM21	21D non-mycorrhizal soybean roots	Non-mycorrhization	3
AM13	13D mycorrhizal soybean roots	Early Symbiosis	3
AM21	21D mycorrhizal soybean roots	Middle Symbiosis	3
AM26	26D mycorrhizal soybean roots	Late Symbiosis	3

Table S2 Expression levels and AM induction of candidate *GmGELPs* during soybean AM symbiosis.

Gene name	NM21	AM13	AM21	AM26	Log_2FC (AM21vsNM21)
GmGELP6	0.067063	105.0945	330.589	236.9129	12.137
GmGELP29	0	67.37314	98.92971	81.44888	Inf
GmGELP140	0.057028	14.76267	35.69567	29.53782	9.236
GmGELP141	0.024889	60.29358	182.5035	125.5304	12.746
GmGELP151	0.275545	29.82472	130.497	130.2133	8.8157

Expression values are shown as FPKM. log_2FC values were calculated from the AM21 versus NM21 comparison. “Inf” indicates that expression was not detected in NM21 but was induced under AM conditions. *GmGELP6*, *GmGELP29*, *GmGELP141*, and *GmGELP151* were prioritized based on strong AM induction and relatively high expression levels. *GmGELP140* was included because it showed an AM-associated expression pattern and was closely related to *GmGELP141*, although its expression level was lower than those of the other candidates.
